# Malnutrition-related parasite dissemination from the skin in visceral leishmaniasis is driven by PGE_2_-mediated amplification of CCR7-related trafficking of infected inflammatory monocytes

**DOI:** 10.1371/journal.pntd.0011040

**Published:** 2023-01-11

**Authors:** E. Yaneth Osorio, Ashanti Uscanga-Palomeque, Grace T. Patterson, Erika Cordova, Bruno L. Travi, Lynn Soong, Peter C. Melby

**Affiliations:** 1 Department of Internal Medicine, University of Texas Medical Branch, Galveston, Texas, United States of America; 2 Department of Microbiology and Immunology, University of Texas Medical Branch, Galveston, Texas, United States of America; 3 Center for Tropical Diseases and Institute for Human Infection and Immunity, University of Texas Medical Branch, Galveston, Texas, United States of America; 4 Department of Pathology, University of Texas Medical Branch, Galveston, Texas, United States of America; Institute of Postgraduate Medical Education and Research, INDIA

## Abstract

People are infected with *Leishmania donovani* when the parasite is deposited in the dermis during the blood meal of the sand fly vector. Most infected people develop a subclinical latent infection, but some develop progressive visceral leishmaniasis. Malnutrition is a risk factor for the development of active VL. We previously demonstrated increased parasite dissemination from the skin to visceral organs in a murine model of malnutrition. Here we investigated the mechanism of early parasite dissemination. After delivery of *L*. *donovani* to the skin, we found enhanced capture of parasites by inflammatory monocytes and neutrophils in the skin of malnourished mice. However, parasite dissemination in malnourished mice was driven primarily by infected inflammatory monocytes, which showed increased CCR7 expression, greater intrinsic migratory capacity, and increased trafficking from skin to spleen. PGE_2_ production, which was increased at the site of skin infection, increased monocyte CCR7 expression and promoted CCR7-related monocyte-mediated early parasite dissemination in malnourished mice. Parasite dissemination in monocytes was reduced by inhibition of PGE_2_, knockdown or silencing of CCR7 in monocytes, and depletion of inflammatory monocytes through administration of diphtheria toxin to CSFR1-DTR transgenic mice that have monocyte-specific DT receptor expression. CCR7-driven trafficking of infected inflammatory monocytes through the lymph node was accompanied by increased expression of its ligands CCL19 and CCL21. These results show that the CCR7/PGE_2_ axis is responsible for the increased trafficking of *L*. *donovani*-infected inflammatory monocytes from the skin to the spleen in the malnourished host. Undernutrition and production of PGE_2_ are potential targets to reduce the risk of people developing VL. Nutritional interventions that target improved immune function and reduced PGE_2_ synthesis should be studied in people at risk of developing VL.

## Author summary

This study established that inflammatory monocytes contribute to the visceralization of *L*. *donovani* parasites in the malnourished host. We found that the prostaglandin E_2_ produced during the first 72 hours of infection amplified the parasite burden at the site of skin infection and induced a high expression of CCR7 receptor in inflammatory monocytes. CCR7-bearing inflammatory monocytes carried *L*. *donovani* parasites via enhanced migration from the skin infection site to the viscera of malnourished mice. This dissemination of *Leishmania* parasites was abrogated in CCR7 deficient inflammatory monocytes and in mice treated with a prostaglandin E synthase inhibitor. We conclude that PGE_2_ contributes to monocyte-mediated parasite dissemination through the expression of CCR7 and its ligands in malnourished mice.

## Introduction

Visceral leishmaniasis (VL), caused by the intracellular protozoan parasite *L*. *donovani* or *L*. *infantum* (= *L*. *chagasi*), is one of the “Neglected Tropical Diseases” that impacts the poorest regions of the world [1]. It causes about 0.7 million cases per year [2] and is second only to malaria as a cause of death by a parasitic infection. It places ninth among global infectious diseases [3] in disability-adjusted life years (DALYs).

*L*. *donovani* is transmitted to humans when infectious metacyclic promastigotes are deposited in the dermis during the blood meal of the sand fly vector. This inoculation usually does not lead to an overt inflammatory skin lesion, with exception of Post-Kala-azar dermal Leishmaniasis and rare cutaneous forms of *L*. *donovani* [4]. The majority of people who are infected develop a latent infection without signs or symptoms of clinical disease. The innate immune response that occurs within a few hours/days of infection and the long-term adaptive Th1 cellular immune response both play a critical role in preventing the development of active disease [5]. In approximately 10% of infected individuals, the inoculated parasite disseminates from the skin to involve the liver, spleen, and bone marrow and causes the clinical syndrome of fever, cachexia, massive splenomegaly, pancytopenia, and ultimately death. The reason that only a minority of infected individuals develop the full-blown disease, and the mechanism of parasite dissemination, are largely unknown.

Epidemiological and observational studies indicate that undernutrition, which affects nearly one-fifth of the world population [6], has a role in the development of VL. Undernutrition has a plethora of effects on immune function, predisposes to several infectious diseases [7], and is thought to contribute to more than a third of all childhood deaths [6]. The prevalence of undernutrition in patients with active VL is estimated to be 40–95% [8, 9]. Prospective studies document a greatly increased risk for active VL in undernourished individuals [10–13]. Overt undernutrition was also identified as a risk factor for severe disease [11] and death from VL in both children (odds ratio 5.0) and adults (odds ratio 11.0) [14].

Unfortunately, conventional animal models have yielded few insights into the mechanism of parasite dissemination to visceral organs. Immunocompetent mice inoculated in the skin with *L*. *donovani* mimic subclinical infection [15, 16], but parasite dissemination and clinically evident visceral disease is attained only in models that utilize an undernourished host [17–19] or sand fly transmission [20]. The strong association of VL with undernutrition prompted us to investigate *L*. *donovani* infection in a murine model representative of childhood malnutrition. Undernutrition in this model is initiated by a polynutrient-deficient diet [17–19, 21, 22] that includes the superimposed deficiencies of protein, energy, zinc and iron, which are common to the diets of undernourished children [23, 24]. When initiated after weaning, this diet leads to a flat growth curve and biochemical derangements characteristic of children with moderate acute malnutrition [25, 26]. Within 3 days of intradermal *L*. *donovani* infection, the malnourished (MN) mice showed a 7-10-fold increase in parasite dissemination to the liver and spleen [17, 18]. This was associated with reduced lymph node barrier function and altered cell trafficking [17–19].

Our earlier studies demonstrated that lymph node PGE_2_ production correlated with parasite visceralization in malnourished mice [17]. PGE_2_ is an eicosanoid lipid mediator found to be elevated in children with kwashiorkor, and considered to be a potential contributor to immune depression and increased risk of infection [27]. In leishmaniasis, PGE_2_ is induced by sand fly saliva and infection of macrophages [28, 29]. It mediates pro- and anti-inflammatory signals that benefit parasite survival [28, 30]. PGE_2_ also enhances myeloid cell migration [31–34] and suppresses phagocyte function [35].

In the present study, we investigated the mechanism of early parasite dissemination. After delivery of *L*. *donovani* to the skin, we found enhanced capture of parasites by inflammatory monocytes and neutrophils in the malnourished host. Because we found that inflammatory monocytes, but not neutrophils, had a primary role in parasite visceralization, we focused our attention on monocytes. Parasitized monocytes show increased migratory capacity, increased expression of CCR7, and increased trafficking to the spleen. PGE_2_ production at the site of skin infection was increased in the malnourished host and led to increased CCR7 expression on inflammatory monocytes. Early monocyte-mediated parasite capture in the skin and dissemination to viscera was promoted by delivery of PGE_2_ to the site of infection. Early parasite dissemination via inflammatory monocytes was abrogated by blocking PGE_2_ synthesis and knockdown of CCR7 in monocytes in the malnourished host. The significance of inflammatory monocytes in *L*. *donovani* dissemination was further demonstrated in mice with inducible depletion of inflammatory monocytes (CSFR1-LysMcre-DTR mice) which resulted in reduced parasite trafficking through the lymph node and reduced visceral parasite burden after skin infection with *L*. *donovani*.

## Methods

### Ethics statement

The animals used in this study were handled in strict accordance with the recommendations in the Guide for the Care and Use of Laboratory Animals of the National Institutes of Health. The protocol was approved by the Institutional Animal Care and Use Committee of the University of Texas Medical Branch, Galveston, Texas (protocol number 1306027).

### Malnourished and control mice

Age-matched, 3 week-old female Balb/c weanling mice (Envigo) were fed a polynutrient deficient diet (3% protein, 1 ppm zinc and 10 ppm iron, TD.99075, Envigo) or isocaloric, nutrient-sufficient control diet (16.9% protein, 30 ppm zinc and 100 ppm iron (TD.99103, Envigo) as described previously [17, 18]. Food consumption of the control group was measured every 48–72 hrs and the mice in the nutrient-deficient group were fed 90% (by weight) of the food consumed by the control group.

### Infection of mice

After 28 days on the control or nutrient-deficient diet, mice were infected intradermally (ID) in the dorsum of the foot or ear pinna with 10^6^ purified *L*. *donovani* metacyclic promastigotes (IS strain; MHOM/SD/00/S-2D) as described [18]. To trace parasite trafficking in lymph node or spleen, metacyclic promastigotes were labeled with PKH cell trackers (Sigma) or Far Red CellTrace (Invitrogen), washed, and adjusted to 10^6^ per 10–20 μl PBS for infection. Alternatively, *Leishmania* parasites transfected with luciferase gene [36] or fluorescent mCherry [37] were used as specified in the figure legend. Measurement of parasite burden by qPCR was accomplished using primers targeting *Leishmania* rRNA (18S) and the number of parasites was calculated with a standard curve constructed with dilutions of *L*. *donovani* promastigotes as described [38].

### Transgenic mice

Induced deficiency of inflammatory monocytes was studied in a Cre-inducible diphtheria toxin (DTR) expression model (CSFR1-LysMcre-DTR mice). In brief, 6–8 week old female and male offspring were obtained after crossing B6.129P2-Lyz2tm1(cre)Ifo/J (Jackson stock # 004781) with C57BL/6-Tg (Csf1r-HBEGF/mCherry)1Mnz/J (Jackson stock #024046). Carriers of the inducible phenotype (CSFR1-LysMcre-DTR) were identified as described [39]. After being placed on the experimental malnourished diet for 28 days, CSFR1-LysMcre-DTR mice were given 5 ng of Diphtheria Toxin (DT) (Sigma) per gram body weight (approximately 100 ng per mouse) 24h before *L*. *donovani* infection. DT Treatment was repeated every 24h until euthanasia (72h p.i.). Non-carriers (LysMcre) mice injected with PBS or DT and were used as experimental controls.

### Flow cytometry

Cells were obtained of infected mice 24-72h post-ID infection. Tissues were collected in RPMI culture medium containing 10% heat-inactivated fetal bovine serum (FBS) and 1,000 U/mL Penicillin-Streptomycin. The tissue was then treated with collagenase D (2 mg/mL) plus DNAse I (20 μg/mL) in RPMI with 2% FBS for 15 min at 37°C. To isolate skin cells 250 μg/mL Liberase (Roche) was added to the enzyme blend as described. Cells were washed with PBS 0.5% BSA, stained and analyzed by flow cytometry as described [19]. The gating strategy was established following parameters of Rose, et al. [40] and depicted in **S1A–S1D Fig**. The antibodies used for flow cytometry are listed in **S1 Table**. Flow cytometry panels were designed to minimize overlapping fluorescence with the tool Fluorofinder. A Stratedigm (S500) or LSR Fortessa II (BD) flow cytometers were used for sample acquisition. Flowjo V10.6 software was used for flow cytometry analysis. The percentage of infected cells was represented as total percentage of infected cells or percentage of infected cells of the parent population (monocytes, inflammatory monocytes, resident monocytes or neutrophils). The cut-off point of positive populations was established with isotype or FMO controls.

### Transwell cell migration assay

*In vitro* migration assays were performed in 24-well plates containing 5 μm polycarbonate membrane Transwell inserts (Corning). Splenic cells, bone marrow or splenic CD11b^+^ cells were isolated by positive selection with anti-mouse CD11b Magnetic Particles-DM (BD). Purity of myeloid cells expressing CD11b was >85% with this method. Cells (0.125–0.5 x10^6^ cells/100 μL) were placed in the top chamber of the transwell system. Cells migrating to the bottom chamber were recovered after spontaneous migration or in response to CCL21 (R&D systems; 300 ng/mL in the lower chamber). The phenotype and percentage of the cells migrating through the transwell was determined by flow cytometry as above. The absolute number of cells was calculated as percentage of cells determined by flow cytometry x number of cells counted by luminometry (CellTiter Glo, Promega) divided by 100. The luciferase signal relative to number of cells was linear with a correlation coefficient >0.9.

### Labeling of dermal cells

To trace cell trafficking from the skin to the draining popliteal lymph node or spleen, mice were infected in the dorsal skin of the foot and immediately the infection site was painted with 20 μL of 0.5 mM Cell Tracker (Orange CMTMR (5-(and-6)-(((4-chloromethyl) benzoyl) amino) tetramethylrhodamine)) (ThermoFisher Science) as described [18]. Migrating cells that picked up the cell tracer were detected in the lymph node and spleen by flow cytometry.

### Adoptive transfer of monocytes

Bone marrow monocytes (CD11b^+^ cells) were obtained from uninfected or infected malnourished or control mice by selection with 1A8 (Ly6G) (Biolegend or BioXcell) to discard neutrophils, followed by positive selection with CD11b Magnetic Particles-DM (BD). Cells were stained with cell tracers according manufacturer instructions (either 2.5 μM CMTMR, 5 μM CSFE or 1uM Far-Red Cell trace) and washed twice with culture medium. Cells were adjusted to 2.5-4x10^6^ per 10–20 μL and transferred by ID (infection site) or IV injection to control or malnourished mice followed by immediate ID infection with *L*. *donovani*. Cells trafficking to the spleen were identified by flow cytometry at 48 hrs after adoptive transfer.

### *In vivo* inhibition of PGE_2_

To evaluate effect of PGE_2_ on CCR7 expression and parasite dissemination, well-nourished control mice were injected ID with 10 nM of PGE_2_ in 20 μL PBS in the dorsal skin of the foot just prior to infection with 10^6^ PKH-26 red-labeled metacyclic promastigotes. At 48 hrs post-infection, CCR7 expression was determined in uninfected and infected cells by flow cytometry. To evaluate the effect of prostaglandin E_2_ in malnourished mice, we treated malnourished or control mice with a mPGES-1 inhibitor (CAY10526, Cayman Chemical) at 5 mg/kg/day by IP injection in 20 μl DMSO. Control mice were treated IP with 20 μL DMSO vehicle. After 48 hrs of treatment, mice were infected ID with 10^6^ metacyclic promastigotes labeled with PKH-67 green. At 48 hrs post-infection, infected cells and cells trafficking from the infection site to the spleen were determined by flow cytometry and skin painting as above.

### Depletion of neutrophils

Neutrophils were depleted in malnourished mice by injection of 200 μg of anti-Ly6G antibody (1A8) IP once on the day before the infection [41]. Depletion in skin (site of infection) and spleen was confirmed by flow cytometry.

### Prostaglandin E_2_, cytokine production and

For measurement of PGE_2_ production, skin of the infection site and control skin of mice was snap frozen and homogenized at 100mg/mL in PBS containing 10 μM indomethacin. Samples were centrifuged, the protein in the supernatant normalized and PGE_2_ synthase estimated in a competitive assay, which measures the stable derivative Bycyclo Prostaglandin E_2_ (Prostaglandin E2 EIA kit, Cayman).

### Gene expression

Transcripts for cytokines/chemokines and Prostaglandin E synthase (mPGES-1) were quantified by qRT-PCR as described [38]. For qPCR assays fold-change of target genes were calculated with reference to uninfected tissue. The number of parasites was calculated with a standard curve. Primer sequences are listed in **S1 Table**.

### CCR7 knockdown in monocytes

Bone marrow cells from uninfected normal mice were selected with CD11b magnetic beads as above. Cells (10^6^) were suspended in 10 mL RPMI, 10% FBS, without antibiotics and 20 ng/mL GM-CSF for cell viability (>90%). Cells were transfected with Lipofectamine RNAiMAX (Thermo Fisher Sci.) and 20 nM of either predesigned siRNA CCR7 oligonucleotides (Silencer Select, 4390771, locus 12775, Ambion) or control siRNAi oligonucleotides (Silencer Select, 4611, negative control, Ambion). After 72 hrs of transfection, CD11b^+^ cells were selected as above and then labeled with PKH-67 or PKH-26 respectively. PKH labeled cells (10^6^/20 μL) were transferred ID to malnourished mice. Mice were immediately infected ID with 10^6^ metacyclic parasites labeled with 1 μM Far Red cell proliferation tracer (Invitrogen). At 48 hrs after adoptive transfer, cells that migrated from the skin to the spleen and parasite fluorescence were determined by flow cytometry. Data were confirmed in similar transfer experiments with donor monocytes isolated from female CCR7KO mice (Jackson stock # 006621) compared with those of control wild-type (WT) mice with the same C57BL/6J background (Jackson stock # 000664). CCR7KO or WT monocytes (2.5-4x10^6^) were transferred into WT malnourished recipient mice. Infections of recipients of CCR7 KO cells or WT cells were done ID with mcherry *L*. *donovani*.

### Statistical analysis

Data were analyzed with GraphPad Prism 9.0, Software (Graphpad, LLC). Statistical tests were applied according to the normality of the data (Kolmogorov-Smirnov normality test) and software recommendations. All experiments were repeated at least twice. The number of experimental samples and statistical significance is specified in each figure legend. Comparisons between 2 groups were evaluated with two tail Mann-Whitney U test for non-parametric data or two tail unpaired t test for normally distributed data. Comparisons between more than 2 groups were evaluated with Kruskall-Wallis for non-parametric data or ANOVA for normally distributed data with post hoc correction for multiple comparisons. Outliers were identified via the ROUT method with coefficient Q 1% [42] and removed from analysis where appropriate.

## Results

### Early *L*. *donovani* dissemination from the skin contributes to chronic visceral parasite burden

Our previous studies showed that malnutrition promoted early visceralization of *L*. *donovani* to the spleen following inoculation in the skin [17, 18]. To determine if continuous migration of parasites from the infected skin to the spleen augmented chronic visceral infection, we excised either the infected or contralateral uninfected ear pinna of malnourished mice at 72h after intradermal infection. We found no difference in splenic parasite burdens at 21 days post-infection (p.i.) in malnourished mice that had the skin-infection site removed at 72h p.i. compared to control mice that had the uninfected ear removed (**S2A and S2B Fig**). This suggests that parasite visceralization in the first hours of infection is adequate to establish visceral infection and ongoing dissemination of parasites from the skin did not significantly add to the visceral parasite burden.

### Malnutrition amplifies the early dermal parasite burden of *L*. *donovani*

**Fig 1:**
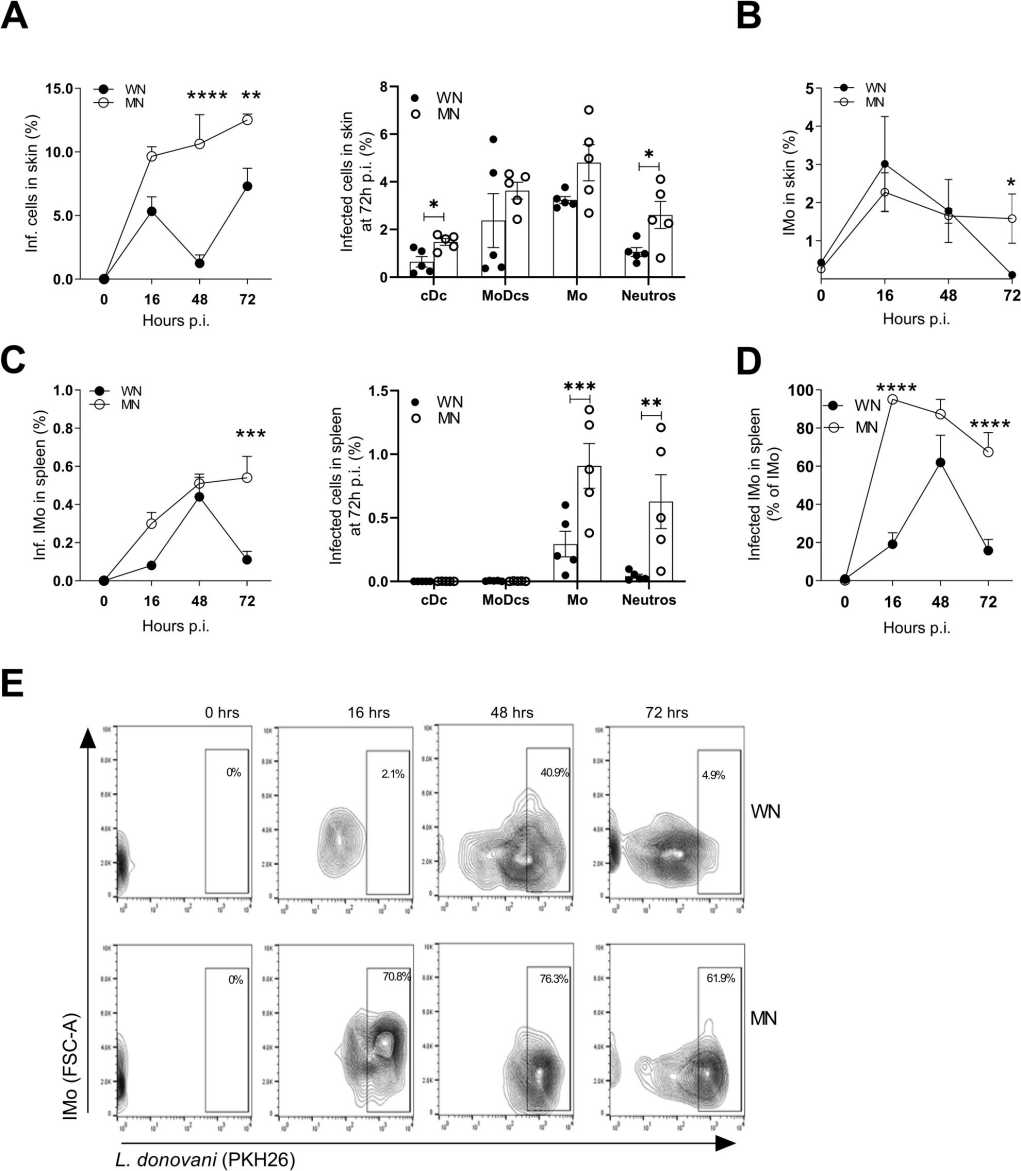
Parasite burden is increased in malnourished mice infected with *L*. *donovani*. **A**. Kinetics of accumulation of infected cells (Left panel) and percent of infected cells including conventional dendritic cells (cDC), monocyte derived dendritic cells (MoDCs), monocytes (Mo), and neutrophils (N) in skin at 72 hr post-infection (Right panel), relative to the total skin cells. **p<0.01, ****p<0.0001 (2-way Anova, Sidak’s multiple comparison test); *p<0.05 (Unpaired t-test). **B**. Kinetics of inflammatory monocyte (Imo) accumulation as percentage of total cells in the skin of WN or MN mice following intradermal *L*. *donovani* infection. *p = 0.05 (Unpaired t-test). **C.** Kinetics of infected inflammatory monocyte (Imo) accumulation showed as percentage of total cells in the spleen of WN and MN mice following intradermal *L*. *donovani* infection (Left panel) and percent of infected cells relative to total spleen cells at 72 hr after intradermal infection with *L*. *donovani* (Right panel). ***p = 0.0001 (2-way Anova). *** *p* = 0.008; **p = 0.014 (2-way Anova). **D.** Percent of infected inflammatory monocytes represented with reference to the parental population (inflammatory monocytes) in the spleen of WN and MN mice following intradermal *L*. *donovani* infection. ****p<0.0001 (2-way Anova). **E**. Representative contour plot gating in inflammatory monocytes (Imo) to show percent infected inflammatory monocytes in spleen. The complete gating strategy is shown in S1A Fig. N = 4–5 mice per time point per group.

To understand the mechanisms responsible for early parasite dissemination, we first studied the site of skin infection. We found accumulation of infected cells in the skin of the MN group **(Fig 1A)**. There was an increase in the proportion of infected total cells, conventional dendritic cells (cDCs), neutrophils, and monocytes in the skin of MN mice between 48 and 72h p.i. **(Figs 1A and S2C)**. The percent of inflammatory monocytes (**Fig 1B)** and the proportion of accumulated infected neutrophils was increased in the skin of MN mice compared with control mice at 72h p.i (**Figs 1A and S3).**

### Malnutrition promotes early accumulation of infected monocytes and neutrophils in the spleen after dermal infection

We performed a time course study from 16–72 hrs post-infection to identify the infected splenic phagocytes early after skin infection, in WN and MN mice. The proportion of infected inflammatory monocytes was increased in spleen of MN mice at 72h p.i. (**Fig 1C**). The proportion of infected monocytes (*p* = 0.0008**)** and infected neutrophils (*p* = 0.0014) were enhanced in the spleen of MN mice compared to control (WN) mice at 72h p.i. **(Fig 1C)**. The majority of splenic inflammatory monocytes **(Fig 1D and 1E)** and neutrophils **(S3B and S3C Fig)** were infected in the MN mice. There were few infected cDCs (WN, 0.1%± 0.1%; MN, 0.04%± 0.002%) and MoDCs (WN, 0.1%± 0.1%; MN, 0.01%± 0.001%) in the spleen, with no difference between the WN and MN groups (**Fig 1C**). The rarity of these infected cells in the spleen indicate that they are not likely to play a significant role in parasite dissemination in the MN host.

### Malnutrition promotes myeloid cell migration and increased trafficking of monocytes from the dermal infection site to spleen

**Fig 2:**
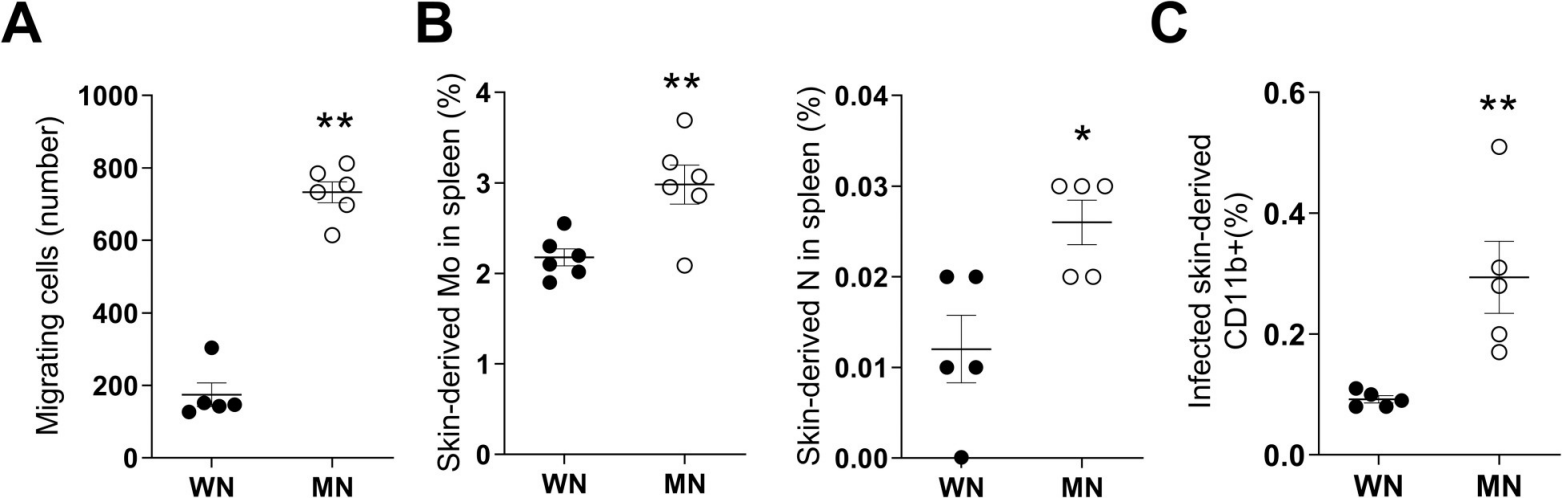
Myeloid cells from malnourished mice have enhanced migratory capacity. **A.** *Ex vivo* spontaneous migration of CD11b+ myeloid cells isolated from bone marrow of 72-hour *L*. *donovani*-infected well-nourished (WN) or malnourished mice (MN) in a 90-minute transwell assay. Shown as number of total migrating cells. *p = 0.0043 (Mann-Whitney test). **B**. Percent of skin-derived monocytes (Mo) in spleen (Left panel) and skin-derived neutrophils (N) spleen (right panel) in 72-hour *L*. *donovani*-infected WN and MN mice. **p = 0.006 (Unpaired t-test); *p = 0.047 (Mann-Whitney test). **C**. Percent of skin-derived infected CD11b+ cells in spleen of WN and MN mice relative to total spleen cells **p = 0.0098 (Unpaired t-test). Data are from a single flow cytometry experiment. N = 5 mice per group.

To establish whether differences in splenic accumulation of infected myeloid cells between WN and MN mice were due to differences in the trafficking of cells from the skin infection site to spleen, we first evaluated the intrinsic migratory capacity of myeloid cells from MN and WN mice. Using an *in vitro* transwell migration assay with myeloid cells isolated from bone marrow of skin-challenged mice, we found that myeloid cells from MN mice had increased spontaneous migration compared to WN mice **(Fig 2A;** *p =* 0.0043**)**. This indicates that even outside of the tissue environment, myeloid cells from the infected MN host have greater spontaneous migration. To evaluate whether this enhanced migration was observed *in vivo*, we topically applied a fluorophore [19, 43] to the skin of WN and MN mice at the time of infection. We found a significant increase in the proportion of skin-derived monocytes **(Fig 2B;** *p =* 0.006**)** and neutrophils **(Fig 2B;** *p* = 0.047**)** that had trafficked to the spleen of MN mice compared to controls. However, the number of monocytes in the spleen that had originated in the skin was approximately 10-fold greater than skin-derived neutrophils **(Fig 2B)**. The relatively minor contribution of neutrophils to early parasite dissemination was confirmed through depletion of neutrophils in malnourished mice prior to infection. Effective depletion of neutrophils was confirmed in the skin and spleen at 24 and 72 hrs post-infection (**S4A and S4C and S4E Fig**). Neutrophil depletion did not have a significant effect on parasite load in the skin at 2, 24, 48, and 72 hrs post-infection (**S4B and S4D Fig**), nor did it change the percent of infected cells in the spleen at 24 or 72 hrs p.i. (**S4B and S4F Fig**). Therefore, we focused our attention for the remainder of the study on inflammatory monocytes (neutrophil data is primarily presented in supplementary figures).

To track parasitized phagocytes from the dermal infection site to the spleen, we labeled *L*. *donovani* metacyclic parasites with PKH before infection and applied the cell tracking fluorophore to the skin of mice following parasite inoculation. Although the proportion of skin-derived infected cells in the spleen was small, there was significantly increased trafficking of infected CD11b+ myeloid cells from the site of skin infection to the spleen in MN mice **(Fig 2C;** *p* = 0.0098**)**.

### Malnutrition-related increased trafficking of infected monocytes from the dermis is driven by changes intrinsic to both the monocytes and the tissue environment

**Fig 3:**
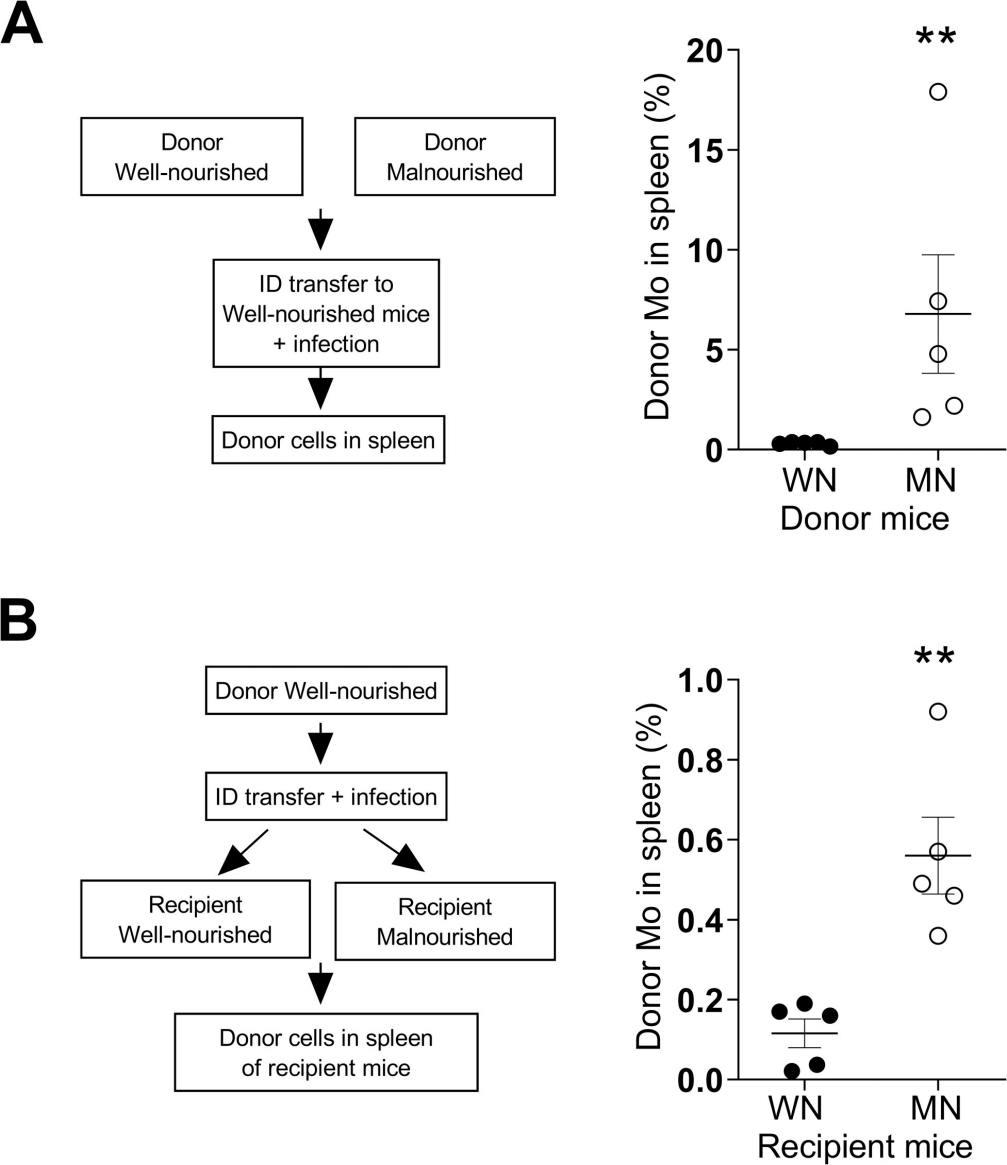
Changes intrinsic to inflammatory monocytes and the tissue environment enhance malnutrition-related trafficking of cells from the dermal infection site to spleen. **A**. Schematic: monocytes from well-nourished (WN) or malnourished (MN) donor mice were labeled with PKH-67 or PKH-26, respectively, and equal numbers of labelled cells were transferred by ID injection to recipient control (WN) mice at the site of simultaneous *L*. *donovani* skin infection. Percentage of transferred monocytes that trafficked to the spleen after ID delivery was determined by flow cytometry. Data shown are from a single experiment that is representative of two independent experiments using 3–5 mice per group. **p = 0.06 (Unpaired t-test). **B**. Schematic: monocytes from control (WN) donor mice were labeled with PKH and transferred to recipient WN or MN mice by ID injection at the site of *L*. *donovani* skin infection. Percent of transferred monocytes that trafficked to spleen after ID delivery in recipient mice. **p = 0.0025 (Unpaired t-test). N = 5 donor mice and N = 5 recipient mice per group.

We performed adoptive transfer experiments to determine if changes intrinsic to monocytes from MN mice favored migration to the spleen. We isolated CD11b^+^ monocytes from bone marrow of MN and WN mice, labeled the cells with a cell tracer (CMTMR), and transferred them to the site of simultaneous dermal *L*. *donovani* infection in control (WN) mice. We found that a higher proportion of monocytes transferred from MN donors trafficked to the spleen compared to monocytes from WN donors **(Fig 3A;** *p =* 0.06**)**. Similar results were obtained when the cells were transferred to the blood by the intracardial route **(S5A Fig)**, suggesting that spleen-delivered signals preferentially enhanced the accumulation of monocytes from MN compared to WN donors. Concordant with the results of *in vitro* migration studies **(Fig 2A)**, these findings indicate that malnutrition alters the intrinsic properties of monocytes that contribute to their increased cell migration and accumulation in the spleen.

To determine if the tissue environment of the malnourished host promotes cell migration and parasite dissemination, we transferred labeled myeloid cells derived from normal control mice to the site of dermal infection in WN and MN mice. More monocytes (from the normal donor) trafficked from the skin to spleen in recipient MN mice than in recipient WN mice **(Fig 3B;** *p =* 0.003**)**. When we tracked cells transferred to the blood by the intracardial route, the results were similar **(S5B Fig;** *p =* 0.02**)**, suggesting that the splenic environment of MN mice promotes increased accumulation of monocytes. Collectively, the cell transfer studies indicate that malnutrition induces changes intrinsic to monocytes and the tissue environment that promotes monocyte trafficking and parasite visceralization to the spleen.

### Prostaglandin E_2_ promotes splenic accumulation of parasitized monocytes following dermal infection

**Fig 4:**
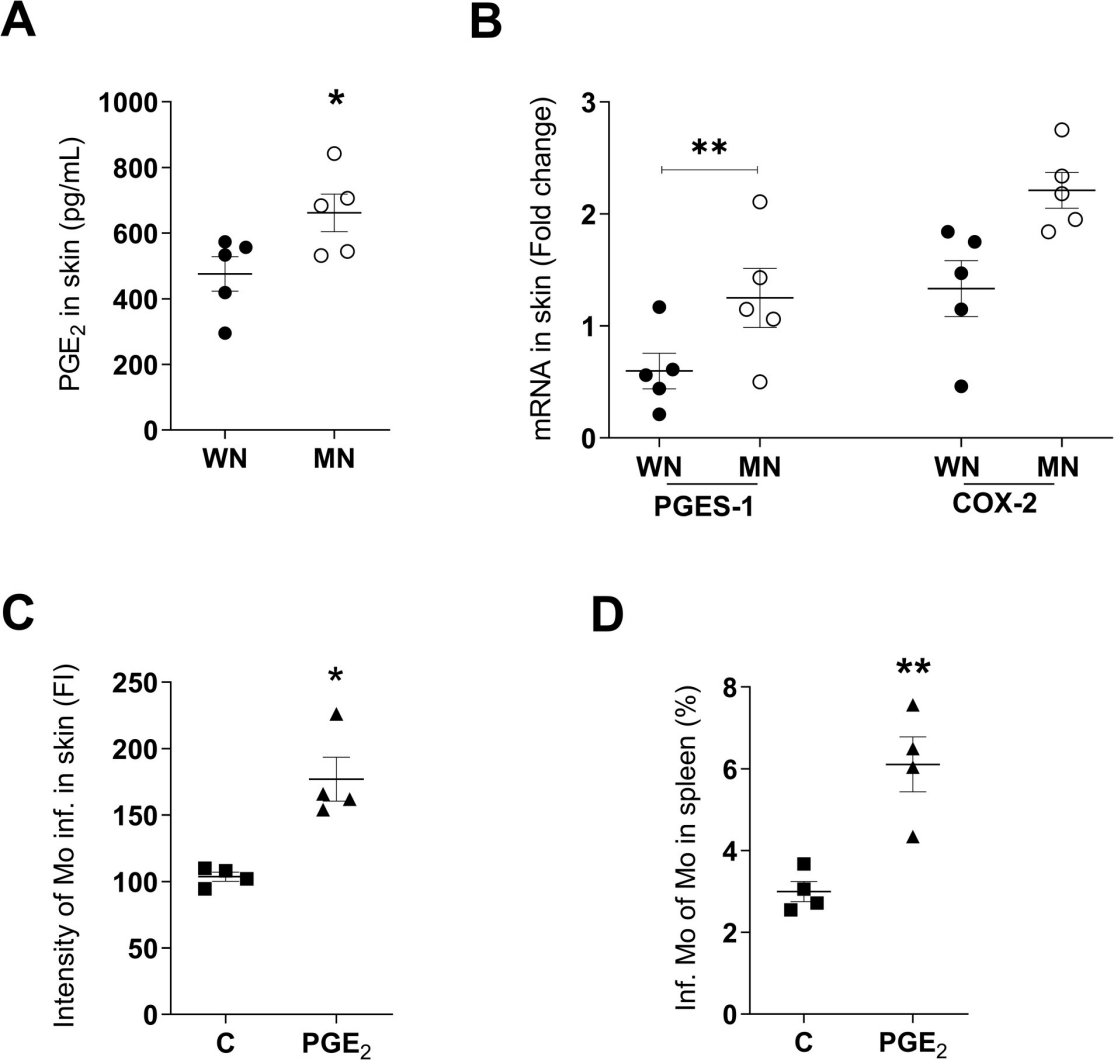
PGE_2_ enhances *L*. *donovani* infection of inflammatory monocytes and their trafficking from skin to spleen. **A**. Prostaglandin E_2_ (PGE_2_) production in skin of control (well-nourished; WN) or malnourished (MN) mice at 24h after *L*. *donovani* infection; determined by competitive ELISA. *p = 0.04 (Unpaired t-test). **B**. PGE_2_ synthase-1 (PGES-1) and cyclooxygenase-2 (COX-2) mRNA expression at site of *L*. *donovani* skin infection in WN and MN mice at 24h post-infection; determined by qPCR. *p = 0.01 (Unpaired t-test). **C**. Intensity of infection (fluorescence intensity from PKH-26 labeled parasites) of skin monocytes from normal (WN) mice infected intradermally with *L*. *donovani* and treated with DMSO (control; C) or PGE_2_, (10 ng per dermal infection site in DMSO). Evaluated at 48h p.i. by flow cytometry. *p = 0.02 (Mann-Whitney test). **D.** Percent of infected monocytes in spleen of normal mice infected intradermally with *L*. *donovani* and treated intradermally with DMSO control (C) or 10 ng PGE_2_ at the time of ID infection. Evaluated at 48h p.i. by flow cytometry. **p = 0.004 (Unpaired t-test). N = 4–5 mice per group.

Our previous studies showed that increased lymph node production of PGE_2_ was associated with parasite dissemination [17], and that macrophages from MN mice produced high levels of PGE_2_ [22]. Other investigators found PGE_2_ to be a potent stimulus of myeloid cell migration [31–34]. Therefore, we considered that PGE_2_ might have a causal role in the increased leukocyte trafficking and parasite dissemination in MN mice. We found increased PGE_2_ in the skin of MN mice compared to control mice 24 hrs after infection with *L*. *donovani* (**Fig 4A**; *p* = 0.04). We also found marginally increased expression of mRNA for COX2 (responsible for the initial step in synthesis of prostaglandins from arachidonic acid; *p =* 0.07) and significantly increased expression of PGE_2_ synthase (PGES-1; responsible for the last step in PGE_2_ synthesis; *p =* 0.01) in the skin of infected MN mice **(Fig 4B)**. To determine if PGE_2_ increased early parasite dissemination, we treated normal (WN) mice with PGE_2_ at the time of infection and evaluated the dermal and splenic parasite burden 48h later. Delivery of PGE_2_ to the site of skin infection significantly increased the intensity of monocyte infection (**Fig 4C**; *p* = 0.02) and proportion of infected monocytes in the spleen (**Fig 4D**; *p =* 0.004). PGE_2_ also induced an increased frequency (*p* = 0.058) and intensity (*p* = 0.0008) of neutrophil infection in the skin **(S6A Fig)**, but did not significantly increase the percent of infected neutrophils in the spleen (**S6B Fig**; *p =* 0.11). This suggests that PGE_2_ amplifies the localized infection but does not directly affect parasite dissemination to the spleen via neutrophils.

### Malnutrition and PGE_2_ enhance *L*. *donovani*-induced CCR7 expression on monocytes

**Fig 5:**
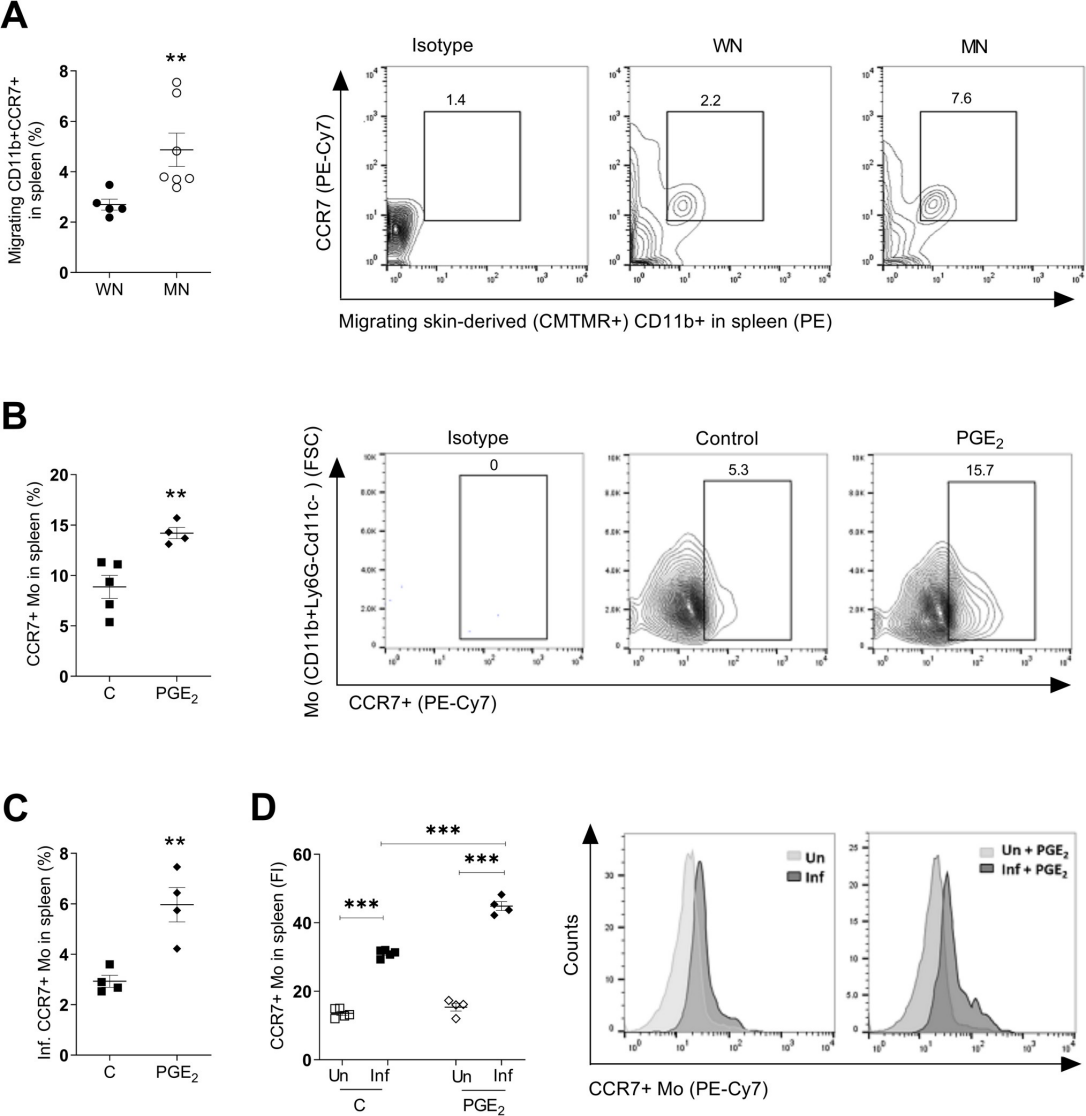
Malnutrition-related increase in CCR7+ skin-derived monocytes in the spleen of mice following intradermal *L*. *donovani* infection is amplified by PGE_2_. **A.** Percent of CCR7+ CD11b+ cells trafficking from skin (skin-derived CMTMR+) relative to total CD11b+ cells in the spleen of well-nourished (WN) or malnourished (MN) mice at 72h after ID infection and skin painting with CMTMR. Shown as a graph (N = 5–7 mice per group) and representative contour plot.**p = 0.02 (Unpaired t-test). **B.** Percent of CCR7+ monocytes relative to total monocytes in the spleen of control (C; DMSO-treated) and PGE_2_-treated mice. Determined by flow cytometry at 48 hrs p.i. Shown as a graph (4–5 mice per group) and representative contour plot. **p = 0.006 (Unpaired t-test). N = 4 mice per group. **C.** Percent of infected CCR7+ monocytes relative to total monocytes in spleen of control (C; DMSO-treated) and PGE_2_-treated mice at 48 hrs after ID infection. **p = 0.005 (Unpaired t-test). N = 4 mice per group. **D.** Intensity of CCR7 expression in monocytes without parasites (Un) or containing parasites (Inf) in spleen of control (C) and PGE_2_-treated mice 48 hrs after ID infection with PKH-26 labeled *L*. *donovani*. ***p<0.0001 (One-way Anova). Shown as a graph (N = 4 mice per group) and representative histogram of 2 independent experiments.

Because PGE_2_ induces CC chemokine receptor 7 (CCR7) expression [31, 32] and CCR7 has a critical role in the migration of myeloid cells from the skin to draining LN [44–47], we evaluated the effect of malnutrition and PGE_2_ on CCR7 receptor expression and its role in cell trafficking from skin to spleen. We found a higher proportion of CCR7-bearing myeloid cells that had trafficked from the site of dermal infection to the spleen in MN compared to WN mice (**Fig 5A**; *p* = 0.02). PGE_2_ treatment of normal (WN) *L*. *donovani* skin-infected mice also increased the percent of CCR7-bearing total splenic monocytes (**Fig 5B**; *p =* 0.006) and infected CCR7-bearing splenic monocytes (**Fig 5C**; *p =* 0.005). Analysis of the fluorescence intensity showed that CCR7 expression was significantly greater in infected splenic monocytes compared to uninfected splenic monocytes (**Fig 5D**; *p*<0.0001), and that CCR7 expression was further enhanced in infected monocytes by exposure to PGE_2_ (**Fig 5D;** *p*<0.0001). Remarkably, the treatment with PGE_2_ increased the proportion of infected monocytes (**S7A Fig**) and infected CCR7-bearing monocytes **(S7B Fig)**, but not cDCs, MoDCs and neutrophils trafficking to the draining lymph node **(S7A and S7B Fig)**. This suggests that the combined effect of infection and PGE_2_ on CCR7 expression in this model is relatively selective for monocytes.

### Monocyte migration and trafficking of parasites to the spleen in malnourished host is regulated by PGE_2_

**Fig 6:**
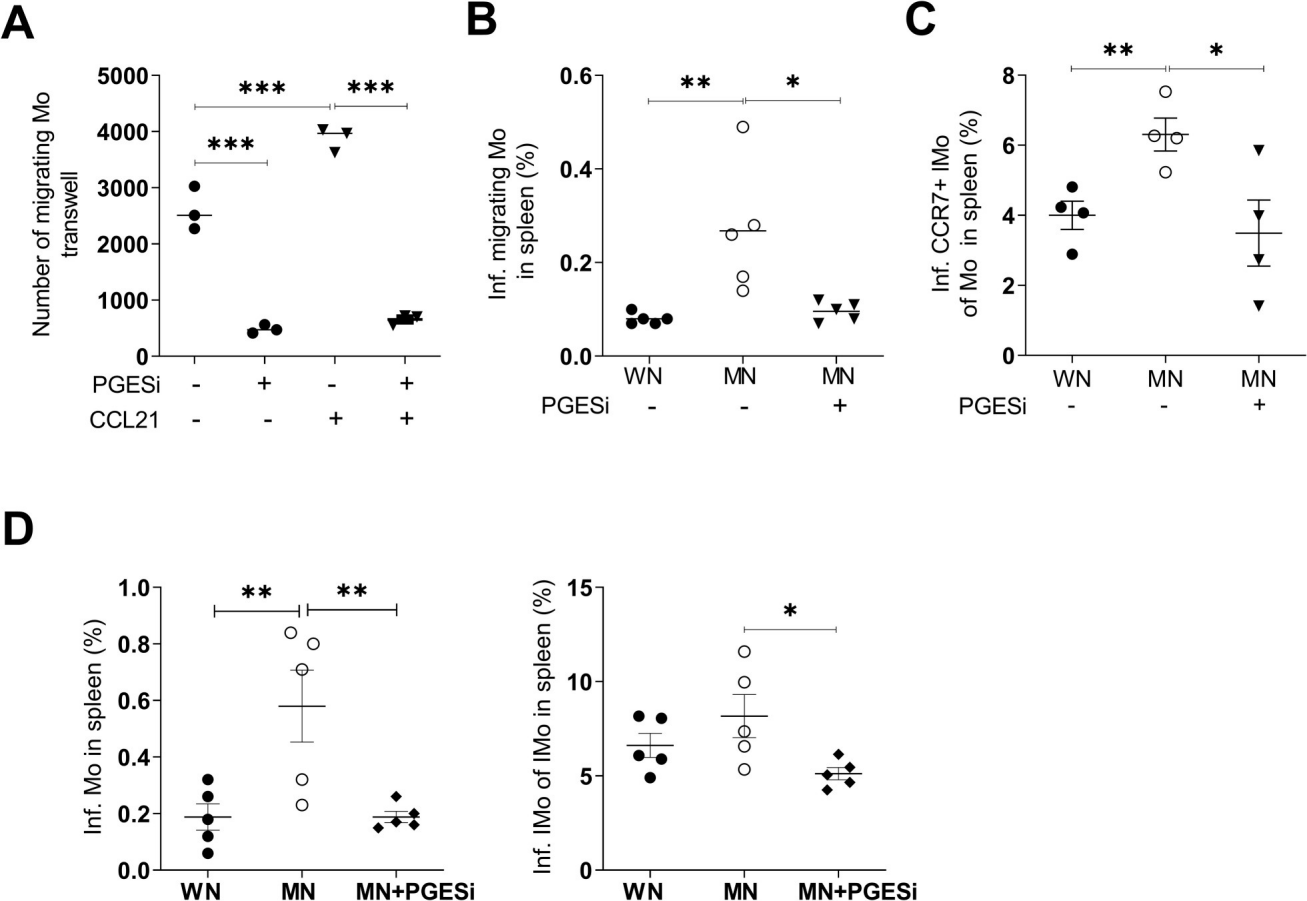
Inhibition of prostaglandin E synthesis blocks the migration of monocytes in malnourished mice. **A.** Migration of monocytes from *L*. *donovani*-infected MN mice with or without treatment with mPGES-1 inhibitor (PGESi, 5mg/kg/IP/day), starting 48h before infection and continuing *ex vivo* at 5 μM, with or without exposure to CCL21 gradient (300ng/mL in lower chamber).***p<0.0001 (One-way Anova). Pooled cells from N = 5 mice per group (triplicate). **B**. Percent of infected skin-derived monocytes in spleen of WN mice and MN mice treated IP with DMSO or MN mice treated with prostaglandin E_2_ synthase inhibitor (PGESi) (CAY10526, 5mg/kg/day).*p = 0.01; **p = 0.08 (One-way Anova). N = 5 mice per group. **C.** Percent of infected CCR7+ inflammatory monocytes in the spleen of WN mice and MN mice treated with DMSO or MN mice treated with PGESi inhibitor. Determined at 48h p.i. by flow cytometry. **p = 0.0098 (Unpaired t-test); *p = 0.037 (One-way Anova). N = 4 mice per group. **D.** Percent of infected monocytes (Left panel) and proportion of infected inflammatory monocytes of inflammatory monocytes (Right panel) of WN mice, and MN mice treated IP with DMSO (MN) or prostaglandin E synthase inhibitor (MN + PGESi, CAY10526, 5mg/kg/day/IP) starting 48 hr before infection. Evaluated at 48h p.i. by flow cytometry. **p = 0.01, *p = 0.04 (One-way Anova). N = 5 mice per group.

To confirm that PGE_2_ influenced cell trafficking, we used an *ex vivo* transwell assay and found inhibition of CCL21-driven (*p*<0.0001) migration of bone marrow monocytes from malnourished mice treated with a PGES-1 inhibitor (**Fig 6A**). Since PGE_2_ treatment of normal mice increased CCR7 expression on monocytes **(Fig 5B and 5C)**, we used the PGES-1 inhibitor to evaluate the link between PGE_2_ and CCR7 expression in monocytes, and monocyte-mediated parasite dissemination. *In vivo* treatment of MN mice with the PGES-1 inhibitor significantly reduced (to control levels) the trafficking of infected monocytes from skin to spleen (**Fig 6B**). We found a reduced proportion of infected inflammatory monocytes that expressed CCR7 in the spleen of malnourished mice treated with the PGE_2_ inhibitor (**Fig 6C**) suggesting that PGE_2_ is regulating CCR7. Collectively, these data show that CCR7-bearing infected inflammatory monocytes accumulate in the spleen after intradermal infection of malnourished mice with *L*. *donovani*, and that PGE_2_ regulates CCR7 expression and trafficking of infected monocytes. Likewise, PGES-1 inhibitor decreased the proportion of infected total monocytes (*p* = 0.01) and infected inflammatory monocytes (*p* = 0.04) in the spleen of MN mice (**Fig 6D**). However, the inhibition of PGE_2_ synthesis did not significantly affect the frequency of infected monocytes or neutrophils in the skin of MN mice **(S8A Fig)**. Inhibition of PGE_2_ synthesis did not significantly reduce the trafficking of infected neutrophils to the spleen **(S8B Fig)**. Collectively, these results suggest that PGE_2_ promotes parasite dissemination via increased trafficking of infected inflammatory monocytes, but not neutrophils, to the spleen. The lack of effect of the PGES-1 inhibitor on skin parasite burden suggests that PGE_2_ is not likely to be the main regulator of skin parasite burden in malnutrition.

### CCR7 enhances parasite dissemination in inflammatory monocytes

**Fig 7:**
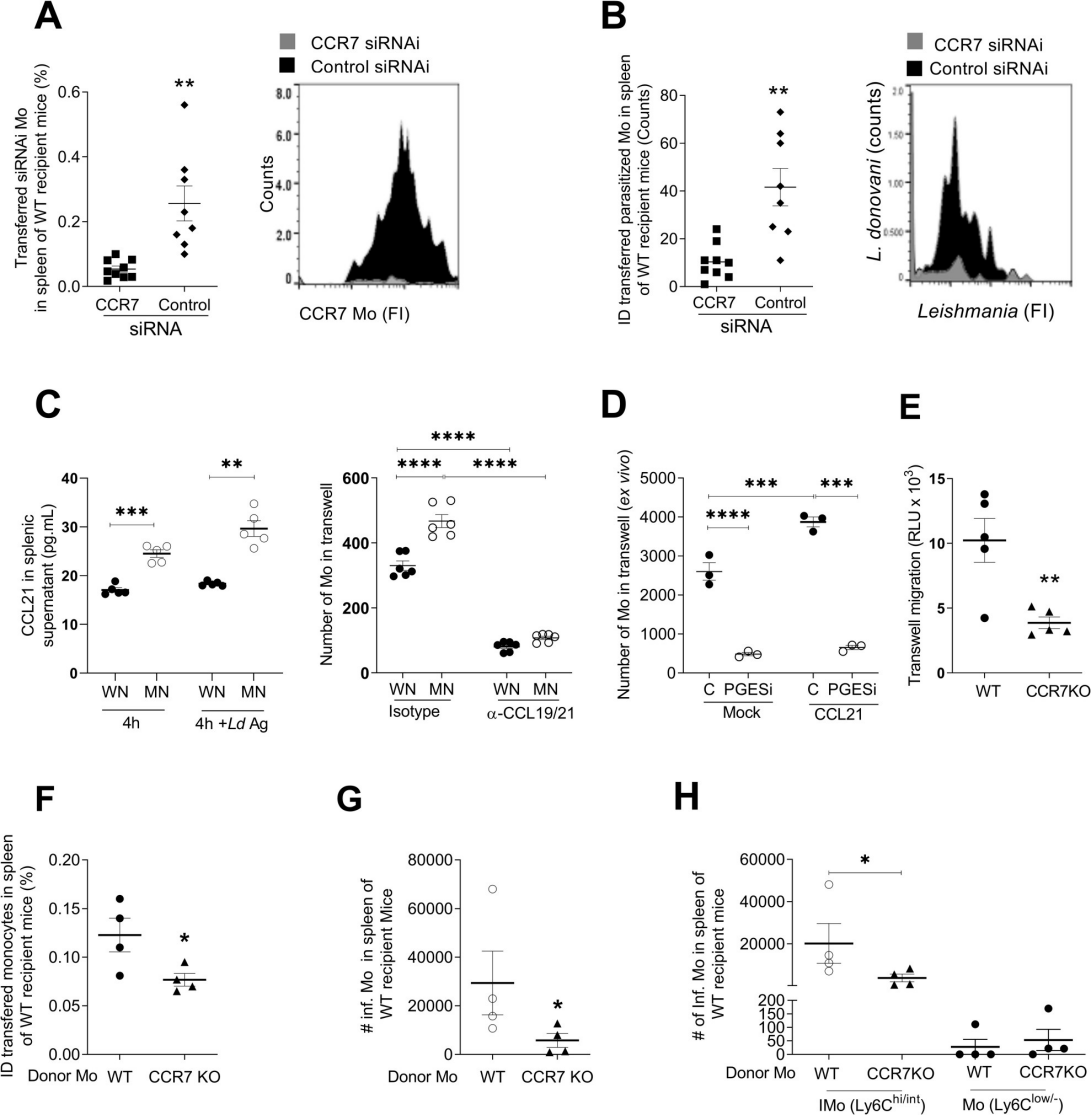
Monocyte-mediated, PGE_2_-driven *L*. *donovani* visceralization is dependent on CCR7 and its ligands in malnourished mice. **A.** Percent of skin-derived monocytes in the spleen of MN mice following intradermal infection with Far-Red-Cell Trace labeled *L*. *do*novani and ID transfer of CCR7-silenced PKH-67-labeled monocytes (CCR7 siRNA) or control PKH-26 labeled monocytes (control siRNA). Determined by flow cytometry 48 hrs after ID transfer and infection; shown as graph and representative histogram.**p = 0.0014 (Unpaired t-test). N = 8–9 mice/group. **B.** Counts of fluorescent parasites (number of events) in control (siRNA control) or CCR7-deficient monocytes (CCR7 siRNA) in the spleen 24 hrs after ID infection and monocyte transfer in MN mice. Determined by flow cytometry with gating on the population of labeled-transferred monocytes. Shown as graph and representative histogram. **p = 0.0011 (Unpaired t-test). N = 8–9 mice per group. **C.** Left panel: Levels of CCL21 released to supernatants of spleen cell cultures from WN or MN mice over 4 hrs culture with or without exposure to *L*. *donovani* antigen (Ld Ag). N = 5 mice per group. Right panel: migration of monocytes in response to splenic chemokines determined in a transwell assay with 10^6^ bone marrow-derived monocytes from control mice in the upper chamber and 24-hr culture supernatants from 10^6^ spleen cells from *L*. *donovani* infected well-nourished (WN) or malnourished (MN) mice in the lower chamber. Monocyte migration to the lower chamber was determined by cell counting and flow cytometry after 16h in the presence of neutralizing anti-CCL19/21 or isotype control antibody. **** *p*<0.0001; ***p = 0.0002; **p<0.001 (One-way Anova). N = 6 mice per group. **D.** *Ex vivo* transwell migration of bone marrow-derived monocytes from *L*. *donovani*-infected malnourished mice treated with DMSO (C) or prostaglandin E synthase inhibitor (PGESi) (5 mg/kg/IP/day, 48h before infection and 5 μM *ex vivo*), in response to buffer alone (Mock) or 300 ng/mL CCL21. Monocyte migration to the lower chamber was determined after 16h of culture by luminometry. Pooled cells from N = 5 mice per group (triplicate). **** *p*<0.0001; ***p = 0.0006 (One-way Anova). **E.** Transwell assay with monocytes from CCR7KO mice or WT mice infected *in vitro* with 1:5 mCherry-*L*. *donovani* promastigotes migrating toward 200 ng/mL CCL21 gradient. Number of monocytes in lower chamber determined by luminometry (CellTiter-Glo). **p = 0.0065 (Unpaired t-test). N = 5 mice per group. **F.** Percent of WT or CCR7KO donor monocytes found in spleen of malnourished recipient mice 48 hr after of intradermal transfer. Identified in the spleen by the fluorescence of a Far-red cell trace and flow cytometry. *p = 0.047 (Unpaired t-test). N = 5 donor mice per group (WT or CCR9 KO) and 8 recipient WT malnourished mice. **G**. Number of infected monocytes in spleen of malnourished mice infected with mCherry-*L*. *donovani* that were recipients of WT or CCR7KO monocytes. *p = 0.057 (Unpaired t-test). N = 5 donor mice per group (WT or CCR7 KO) and 8 recipient WT malnourished mice. **H.** Number of infected monocytes discriminated by the expression of Ly6C (Ly6C^int/hi^, inflammatory monocytes; Ly6C^low/-^, resident non-inflammatory monocytes) in spleen of malnourished mice infected with mCherry-*L*. *donovani* recipient of WT or CCR7KO monocytes. *p = 0.057 (Mann-Whitney test). N = 4 donor mice per group (WT or CCR7 KO) and 8 recipient WT malnourished mice.

To evaluate a causal role of CCR7 in the monocyte-mediated trafficking of parasites from the skin infection site to the spleen, we knocked down the expression of CCR7 in monocytes by siRNA (**S9A Fig**; p<0.0001) and transferred these cells to the dermal infection site in MN mice. Transferred CCR7-silenced monocytes trafficked to the spleen at a much lower rate than (CCR7-sufficient) monocytes transfected with a control siRNA (**Fig 7A**; *p =* 0.001). Similarly, accumulation of parasitized skin-derived monocytes in the spleen of MN mice was impaired when CCR7 was knocked down (**Fig 7B**; *p =* 0.001). In contrast, knockdown of CCR7 in monocytes had no effect on the percent of total or parasitized monocytes in the skin (**S9B Fig)** and there was a trend toward accumulation of monocytes in the draining lymph node (**S9C Fig**). To identify a role for splenic chemokines in the parasite dissemination via infected inflammatory monocytes, we used an *ex vivo* migration assay. We found that CCL21 produced by spleen cells from MN mice induced greater *ex vivo* monocyte migration compared to spleen cells from WN mice (**Fig 7C**; *p*<0.001). This migration was blocked by neutralization of CCL19 and CCL21 (CCR7 ligands) in the spleen cell supernatants (**Fig 7C**; *p*<0.001). The *ex vivo* migration of monocytes from MN mice induced by CCL21 was inhibited by blocking PGE_2_ synthesis (**Fig 7D**; *p*<0.001), further supporting the role for the PGE_2_-mediated regulation of the CCR7 axis in the monocyte-mediated trafficking of parasites to the spleen.

CCR7-mediated trafficking of monocytes from infected skin to spleen was confirmed in experiments with monocytes obtained from CCR7 knockout mice (CCR7 KO). Reduced *ex vivo* migration of CCR7 KO monocytes compared to wild type monocytes from *L*. *donovani* infected mice was confirmed in an *ex vivo* transwell assay with a CCL21 gradient (**Fig 7E**, *p* = 0.0065). Next, we intradermally transferred donor CCR7 KO monocytes or WT monocytes, into WT malnourished recipient mice infected with mcherry-*L*.*donovani*. We examined the proportion of donor monocytes that migrated to the spleen of recipient WT mice. We found lower proportion of monocytes in spleen of mice that received CCR7 KO monocytes compared with those that received WT monocytes (**Fig 7F**, *p* = 0.047). Accordingly, the number of infected monocytes were reduced in the spleen of WT recipients of CCR7 KO monocytes (**Fig 7G**, *p* = 0.057). This difference corresponded to infected inflammatory monocytes (Ly6C^hi/int^) (**Fig 7H**), since resident monocytes (Ly6+^low^/-) were not affected by the absence of CCR7 **(Fig 7H)**. Interestingly, infected inflammatory CCR7 KO monocytes accumulated in the lymph node of recipient mice, whereas the population of resident monocytes was not affected (**S9D Fig**). Collectively, these experiments show that monocyte-mediated parasite visceralization was enhanced by CCR7 expression in inflammatory monocytes and that CCR7 is crucial for monocyte trafficking through the lymph node. Nevertheless, the migration of CCR7-deficient monocytes was not completely abrogated (**Fig 7B and 7G**), suggesting additional CCR7-independent mechanisms of parasite visceralization.

### Depletion of inflammatory monocytes reduces parasite dissemination in malnourished mice

**Fig 8:**
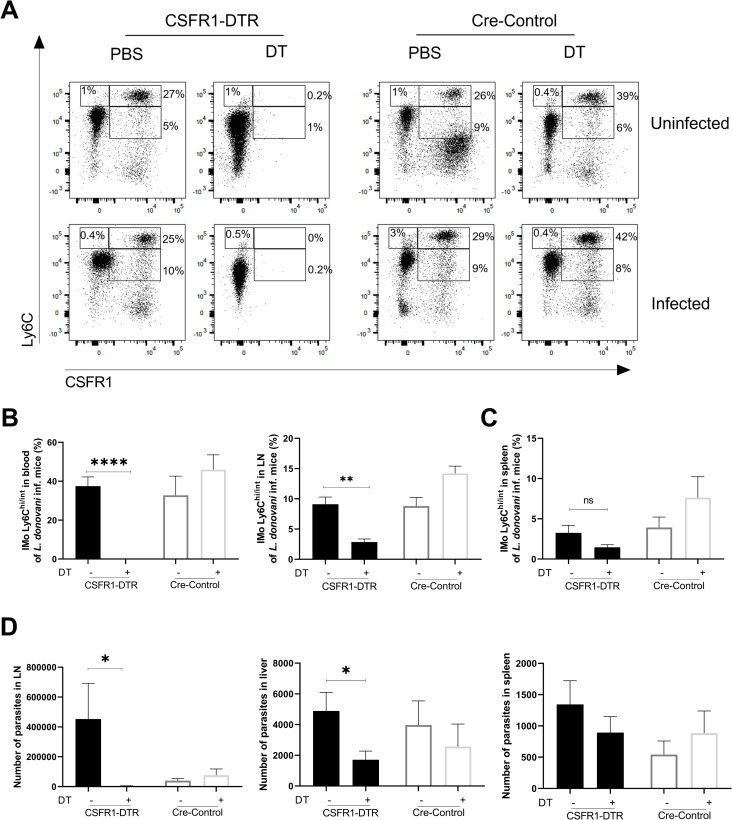
Depletion of inflammatory monocytes reduces early *L*. *donovani* visceralization in malnourished mice. Peripheral blood inflammatory monocytes in malnourished mice transgenic for diphtheria toxin receptor (CSFR1-DTR) and Cre-control malnourished mice were depleted or not by administration of diphtheria toxin (DT) or PBS. **A.** Representative flow cytometry dot-plots showing proportion of inflammatory monocytes in peripheral blood at 72h p.i. with *L*. *donovani* after administration of DT or PBS. N = 7–10 mice per group. **B.** Percent of Ly6C^hi/int^ inflammatory monocytes in peripheral blood and lymph node (LN) of *L*. *donovani*-infected mice with (+) or without (-) administration of DT. ****p<0.0001; **p = 0.0087 (Mann-Whitney test). N = 3–6 mice per group. **C**. Partial depletion of inflammatory monocytes Ly6C^hi/int^ in spleen of *L*. *donovani*-infected mice with (+) or without (-) administration of DT. N = 7–10 mice per group. **D**. Number of parasites (determined by qPCR) in the lymph node (LN), and visceralizing to liver and spleen 72 hr after intradermal *L*. *donovani* infection and depletion of peripheral blood inflammatory monocytes with DT in DTR or Cre-control malnourished mice. LN:*p = 0.046 (Mann-Whitney test); Liver: *p = 0.028 (Unpaired t-test). N = 6–13 mice per group.

We next evaluated the effect of depletion of monocytes on early parasite visceralization in transgenic mice expressing the diphtheria toxin receptor under control of the CD115+ (CSFR1+) promoter (CSFR1-DTR mice). Treatment with DT depleted peripheral blood inflammatory monocytes (Ly6C^hi^) in uninfected (*p* = 0.017) and infected mice (**Fig 8A**; *p*<0.0001). A fraction of Ly6C^int^ inflammatory monocytes and resident monocytes (Ly6C^low/-)^ expressing CD115 was also depleted in peripheral blood by the DT treatment (**Fig 8A** and **S2 Table**). The inflammatory monocytes and parasite dissemination to the draining lymph node was significantly decreased in CSFR1-DTR mice (**Fig** 8B,D; *p* = 0.0087). Parasite visceralization to the liver was also significantly decreased in CSFR1-DTR mice treated with DT compared to Cre-control mice (**Fig 8D**; *p* = 0.028). Together these data support a role for inflammatory monocytes in *L*. *donovani* visceralization through the lymph node. The depletion of inflammatory monocytes in the spleen of CSFR1-DTR mice was partial (**Fig 8C**) and not sufficient to significantly affect the parasite visceralization to the spleen (**Fig 8D**), possibly because a population of CD115-negative splenic inflammatory monocytes escaped DT-mediated depletion (**S2 Table**).

## Discussion

Infection with *L*. *donovani*, the cause of visceral leishmaniasis, is acquired in nature through intradermal delivery of the parasite by the sand fly vector. Most infected individuals develop a subclinical infection, and few develop overt disease. For the parasite to cause visceral disease, it must circumvent the early host defense in the skin and draining LN to disseminate to the visceral organs. The mechanisms through which it does this are unknown. Cutaneous *L*. *donovani* infection in immunocompetent mice is contained in the skin and draining LN without development of overt VL [15, 16]. The established association of malnutrition with development and progression of active VL prompted us to investigate this relationship in a murine model of moderate acute malnutrition. In previous studies we recapitulated the malnutrition-VL association by demonstrating increased early parasite visceralization from the skin in malnourished mice [17, 18]. In this study, we establish a mechanistic underpinning of parasite visceralization from the skin. Malnutrition amplified the cutaneous inflammatory response to *L*. *donovani*, by increased expression of PGE_2_, greater influx of inflammatory monocytes and neutrophils, and increased parasite burden. Parasite dissemination in MN mice was driven primarily by infected inflammatory monocytes. Changes intrinsic to monocytes and changes in the tissue environment combined to promote parasite dissemination in the malnourished host. Increased monocyte-mediated parasite visceralization in malnourished mice was significantly related to CCR7 expression in inflammatory monocytes and PGE_2_ production.

The early cutaneous response to *L*. *donovani* is fundamentally consequential because dissemination of the parasite from the skin ultimately leads to fatal visceral infection. We found that 3 days of skin infection was sufficient to establish chronic visceral infection. We identified several features of the initial skin response that contribute to early parasite visceralization. First, amplified expression of PGE_2_ in the skin of malnourished mice was accompanied by greater influx of myeloid cells. Early recruitment of inflammatory monocytes and neutrophils is consistent with findings in other models of *Leishmania* skin infection [48–53]. Second, increased parasite capture in the skin and impaired effector mechanisms in the malnourished host [7, 17, 18] provide an early means to shuttle parasites to the visceral organs within an intracellular “safe haven”. Whether the “Trojan Horse” phenomenon, in which apoptotic parasitized neutrophils or free parasites that have escaped apoptotic cells [48, 53–55] are subsequently captured by inflammatory monocytes and disseminate, remains to be determined. Lastly, the malnutrition-related increased trafficking of inflammatory monocytes to skin and from skin to spleen suggests that the ingress of cells into the skin was matched by increased egress from the infection site. *L*. *donovani* infected monocytes accumulated in the spleen of MN mice early after skin infection. Depletion of monocytes by delivery of diphtheria toxin to transgenic mice with monocyte-specific DT receptor expression supported the role of monocytes in parasite visceralization to the liver and through the lymph node. However, these approaches have a limited impact in the dissemination of *L*. donovani to the spleen which can be explained by remnant CD115- inflammatory monocytes which carried the parasite.

The role of CCR7 in infected monocyte migration through the lymph node to reach the spleen was further supported by impaired egress and accumulation of infected CCR7 KO inflammatory monocytes in the lymph node. As well by the increased lymph node transit of infected CCR7+ monocytes in mice treated with PGE_2_. Other investigators also demonstrated enhanced trafficking of inflammatory monocytes [56, 57] and neutrophils [58, 59] to LNs under inflammatory conditions, and that these cells can transport pathogens to distant sites [60, 61]. In agreement with these observations, the elimination of peripheral blood inflammatory monocytes in CSFR1-DTR mice significantly decreased the dissemination of parasites to the lymph node and liver, demonstrating the relevance of inflammatory monocytes in *L*. *donovani* visceralization. Changes intrinsic to the infected monocyte population and tissue environment both contributed to the dissemination of parasite-bearing inflammatory monocytes. The finding of a low proportion of migrating infected DCs in the spleen of MN host, indicates that DCs have a minimal role in parasite dissemination to the spleen. This may reflect less parasite phagocytosis by dermal DCs ([51] and our data), impaired differentiation of inflammatory monocytes to DCs in the MN host, and/or identification of the migrating cells as neutrophils due to acquisition of neutrophil surface markers following efferocytosis [52].

While our data indicate that PGE_2_ enhances intrinsic migratory capacity of naïve monocytes in malnourished mice and trafficking of infected monocytes, it could also contribute to parasite dissemination through effects on the tissue environment, as it does to promote metastasis of cancer cells [62–64]. Furthermore, PGE_2_ contributes to visceral disease through suppression of parasite killing capacity in monocytes and macrophages [28–30], either in the skin or spleen. In addition to *L*. *donovani* infection (our data and [65, 66]), protein-energy malnutrition or zinc deficiency [27] also promote increased PGE_2_ production. In contrast to the visceralizing *L*. *donovani*, *L*. *major*, which causes localized skin infection, did not induce COX-2 and PGE synthase in human macrophages [65]. In the setting of natural transmission, sand fly factors are also likely to induce PGE_2_ production by monocytes [28, 67, 68], further promoting infection.

CCR7 and its ligands are known to be crucial for the lymphatic migration of myeloid cells [31, 43, 44, 46]. Several lines of evidence suggest they have a central role in monocyte-mediated dissemination of *L*. *donovani* in the malnourished host. First, we found that CCR7 expression was increased in infected dermal inflammatory monocytes that trafficked to the spleen. Second, monocytes from malnourished infected mice responded to spleen cell supernatants with greater migration, which was abrogated by neutralization of CCL19/21. We previously demonstrated these chemokines to be highly expressed in the spleen of MN mice early after skin infection [19]. Third, monocytes transferred to the site of intradermal infection in MN mice had diminished trafficking from infected skin to spleen when CCR7 was knocked-down or genetically ablated.

Monocyte CCR7 expression was driven by PGE_2_, consistent with findings in other systems [31, 32]. Infection of inflammatory monocytes with *L*. *donovani* induced PGE_2_ production and upregulated CCR7 expression, which was blocked by inhibition of PGE_2_ synthesis. Dissemination of parasite-carrying, skin-derived monocytes, was amplified by exposure to PGE_2_. *Ex vivo* myeloid cell migration and *in vivo* trafficking of parasite-bearing inflammatory monocytes was blocked by inhibitors of PGE_2_ synthesis. This regulation is functionally significant in that inhibition of PGE_2_ synthesis blocked the *ex vivo* migration of monocytes toward CCL21. *L*. *donovani*-induced synthesis of PGE_2_ [66, 69], is likely to trigger expression of CCR7 in infected monocytes through an autocrine effect. However, there are likely other contributors to parasite dissemination in the malnourished mice since we observed that splenic infection was not completely abrogated when CCR7-deficient monocytes were transferred to the dermal site of infection. Collectively, these data indicate that parasite-induced production of PGE_2_, which is part of an exaggerated inflammatory response in the malnourished host [70, 71], amplifies CCR7-dependent trafficking of parasite-bearing inflammatory monocytes to the visceral organs. In contrast, PGE_2_-mediated enhancement of the infection in neutrophils was localized and did not influence *L*. *donovani* dissemination. This was further supported by finding that neutrophil depletion did not significantly affect the parasite visceralization.

In summary, we envision a multistep process following inoculation of *L*. *donovani* in the skin that leads to increased risk of visceral disease in the malnourished host. Malnutrition, which is prevalent in VL-endemic regions, promotes an increased influx and parasite capture by myeloid cells. During natural infection the acute inflammation is enhanced by tissue damage from sand fly probing and release of inflammatory stimuli from the sand fly [20, 48]. Both parasite and sand fly factors trigger PGE_2_ production which is amplified by malnutrition. Following recruitment to the inoculation site, inflammatory monocytes phagocytose parasites and upregulate CCR7 through an autocrine or paracrine effect of PGE_2_. CCR7-dependent trafficking of infected inflammatory monocytes through the lymph node is driven by increased expression of the CCR7 and the ligands CCL19 and CCL21. Consistent with our finding, recent work demonstrated that inflammatory monocytes have a pathologic role in VL by promoting parasite survival in the liver and spleen [72]. On top of this, malnutrition impairs innate and adaptive immune function [7, 73], which leads to impaired parasite killing [74, 75], and disease progression [76, 77]. Nutritional interventions that target improved immune function and reduced PGE_2_ synthesis should be studied in malnourished population at risk of developing VL.

## Supporting information

S1 Table Antibodies and PCR primers. (DOCX)

S2 Table Depletion of monocytes in the spleen of CSFR1-DTR mice. (DOCX)

S1 Fig Gating strategy to identify infected myeloid cell populations in well-nourished and malnourished mice. **A.** Gating strategy to identify parasite infected neutrophils and monocytes. Cells were identified by FSC and SSC properties (A1) and sub-gated in singlets by FSC-A vs. FSC-H (A2). Singlets were sub-gated to exclude dead cells, T cells (CD3+), and B cells (B220+), all in the FITC dump channel (A3). The CD11b+ (APC-cy7) population were sub-gated according to the expression of Ly6C+ (PE-Cy7) in monocytes (CD11b^+^Gr1^low/-^Ly6C^+^) or Gr-1 (PE-cy5) in neutrophils (Cd11b+Gr1^hi^ Ly6C^-/int^) (A4). Monocytes were further classified according the expression of Ly6C as resident monocytes (Ly6C^low/-^) or inflammatory monocytes (Ly6C^hi+/int^) (A5). All populations were sub-gated based the fluorescence of *Leishmania* labeled with PKH26 and FSC-A to identify infected cells (Leishmania PKH26+) (A6). **B.** Gating strategy to identify cDCs and MoDCs. Cells were identified by FSC and SSC properties (B1). Cells were sub-gated in singlets by FSC-A vs. FSC-H (B2). Singlets were sub-gated to exclude dead cells together with T cells (CD3+) and B cells (B220+) in the FITC dump channel (B3). After excluding NK cells (PE-cy7), cells were subgated to identify cDcs (CD11c APCcy7+ CD11b PEcy5-) and MoDcs (CD11c APC-cy7+ CD11b PE-cy5+) (B4). All populations were sub-gated based the fluorescence of the parasite labeled with PKH26 and FSC-A to identify infected cells (Leishmania PKH26+) (B5). **C.** Gating strategy to identify CCR7+ monocytes. After gating singlets in the cell population (C1) neutrophils were identified as Ly6G+CD11b+ cells (C2). Negative cells were sub-gated to identify dendritic cells (CD11c+CD11b-), monocyte-derived dendritic cells (CD11b+CD11c+) or monocytes (CD11b+CD11c-) (C3). Monocytes were sub-gated to identify CCR7+ *Leishmania*+ cells (C4). **D.** Gating strategy to identify inflammatory monocytes in CSFR1-DTR mice. Singlets were sub- gated as above (D1, D2). Dead cells (eF455 positive) were excluded (D3). T cells, NK cells were excluded in the dump channel (D4). Leukocytes (CD45+ positive) were sub-gated (D5 and neutrophils were excluded as CD11b+Ly6G+ cells (D6). No-neutrophils in D6 were sub-gated to identify monocytes as CD11b+CD11c- (D7). These monocytes were sub-gated to identify Inflammatory monocytes (Ly6C^hi/int^) expressing CD115 (CSFR1) (D8).
(TIF)

S2 Fig Impact of early visceralization on chronic visceral *L*. *donovani* load. **A,B**. Visceralization of *L*. *donovani* in well-nourished (WN) mice or malnourished mice (MN) after early surgical excision of the infected ear pinna (Inf) or contralateral uninfected ear pinna (Con) at 72h p.i. Determined at 21 days after intradermal infection by expression of *Leishmania* kDNA in spleen tissue (qPCR) and Leishmania limiting-dilution culture. *p = 0.017; **p = 0.0021, (Kruskal-Wallis test). N = 8 mice per group. **C.** Percent of infected cells including conventional dendritic cells (cDC), monocyte derived dendritic cells (MoDCs), monocytes (Mo), and neutrophils (N) in skin and spleen at 48 hr post-infection relative to the total cells. Skin: *p = 0.03 (Mann-Whitney test, N = 5 mice per group).
(TIF)

S3 Fig Kinetics of neutrophil accumulation in skin and spleen of malnourished mice infected intradermally with *L*. *donovani*. **A**. Kinetics of neutrophil accumulation in the skin of well-nourished (WN) or malnourished (MN) mice following intradermal *L*. *donovani* infection. *p = 0.033 (Unpaired t-test at 72h p.i.). N = 5 mice per group. **B.** Kinetics of neutrophil accumulation in the spleen of WN or MN mice presented as the percent of neutrophils relative to all spleen cells (Left panel), percent of infected neutrophils relative to all spleen cells (Middle panel), and percent of infected neutrophils relative to total splenic neutrophils (Right panel). *p = 0.040; **p = 0.014; ****p<0.0001 (2-way Anova). N = 5 mice per group. **C.** Representative contour plot gated in forward scatter (FSC) showing percent of *Leishmania*-infected neutrophils (CD11b+1A8+) relative to total splenic neutrophils. The full gating strategy is shown in S1A Fig.
(TIF)

S4 Fig Depletion of neutrophils in malnourished mice does not affect visceralization of *L*. *donovani*. **A.** Reduced percent of neutrophils (N) in the skin and spleen (relative to total cells) of malnourished mice treated with the isotype control (MN) or with 200 μg of neutralizing anti-Ly6G antibody (MN+αLy6G) given by the intraperitoneal route the day before ID infection with *L*. *donovani*-LUC. Determined at 24 hrs p.i. **p = 0.014; ***p = 0.0006 (Unpaired t-test). **B.** Parasite burden in the skin infection site and spleen at 24h post-intection. Determined by luminometry. **C.** Reduced percent of neutrophils (N) in the skin (relative to total cells) of malnourished mice treated with the isotype control (MN) or with 200 μg of neutralizing anti-Ly6G antibody (MN+αLy6G) given by the intraperitoneal route the day before ID infection with *L*. *donovani*-LUC. Determined at 72 hrs p.i. ***p = 0.041 (Unpaired t-test). **D.** Left panel: Skin parasite burden in malnourished mice treated with the isotype control (MN) or with 200 μg of neutralizing anti-Ly6G antibody (MN+αLy6G) given by the intraperitoneal route the day before ID infection with *L*. *donovani*-LUC. Determined *in vivo* at 2–72 hrs p.i. Right panel: Percentage of infected cells found in skin after 72 hrs of infection and treatments as described above. **E.** Reduced percent of neutrophils in spleen (relative to toal cells) at 72h of ID infection with *L*. *donovani*. ***p = 0.0001 (Unpaired t-test). **F**. Percent of infected cells found in the spleen determined by flow cytometry. N = 5 malnourished mice per group.
(TIF)

S5 Fig Changes intrinsic to inflammatory monocytes and the tissue environment enhance malnutrition-related trafficking of inflammatory monocytes from the skin infection site to spleen. **A.** Schematic: bone marrow-derived monocytes from donor WN or MN mice were labeled with PKH-67 or PKH-26, combined in equal numbers, and transferred by intracardial (IC) injection to recipient control mice infected ID at the same time with *L*. *donovani*. Labeled monocytes that trafficked to spleen after IC delivery were enumerated by flow cytometry and data are expressed as percent of labeled monocytes relative to total splenic monocytes. *p = 0.052 (Unpaired t-test). N = 5 donor mice per group and 5 recipient mice. **B.** Schematic: bone marrow-derived monocytes from donor well-nourished control mice were labeled and transferred IC to recipient WN or MN mice infected ID at the same time with *L*. *donovani*. Labeled monocytes that trafficked to spleen after IC delivery were enumerated by flow cytometry and data are expressed as percent of labeled monocytes relative to total splenic monocytes. Data shown are from a single experiment that is representative of two independent experiments using 3–5 mice per group. *p = 0.029 (Unpaired t-test). N = 5 donor mice and 3 recipient mice per group.
(TIF)

S6 Fig PGE_2_ enhances skin infection of neutrophils but not the dissemination of parasites in neutrophils. **A.** Percent of infected neutrophils (N) (Left panel), and intensity of infection (fluorescence intensity of parasites; right panel) of neutrophils in mice injected ID with DMSO (vehicle control; C) or PGE_2_ (10 ng in DMSO) followed immediately by ID infection with fluorescent-*L*. *donovani*. *p = 0.058; ***p = 0.0008 (Unpaired t-test). **B.** Percent of infected neutrophils in spleen of intradermally infected control mice (C) or mice treated ID with 10 ng PGE_2_ at the time of ID infection. Evaluated at 48h p.i. by flow cytometry. N = 4 mice per group.
(TIF)

S7 Fig PGE_2_ augments the proportion of infected monocytes expressing CCR7 in lymph nodes. **A.** Proportion of infected monocytes (Mo), conventional dendritic cells (cDCs), monocyte derived dendritic cells (moDCs) and neutrophils in lymph node (LN) of DMSO (C; vehicle control) or PGE_2_ (10 ng in DMSO) in ID treated mice at the time of ID infection with fluorescent-*L*. *donovani*. *p = 0.028 (Mann-Whitney test). **B**. Proportion of infected CCR7-expressing monocytes, cDCs, MoDcs, and neutrophils in LN of DMSO (C; vehicle control) or PGE_2_ (10 ng in DMSO) in ID treated mice at the time of ID infection with fluorescent-*L*. *donovani*. Evaluated by flow cytometry at 48h post-infection. Representative of 2 independent experiments. *p = 0.028 (Mann-Whitney test). N = 4 mice per group.
(TIF)

S8 Fig Prostaglandin E_2_ (PGE_2_) synthesis promotes parasite capture by neutrophils in skin of infected mice. **A.** Proportion of infected monocytes and infected neutrophils in skin of well-nourished (WN) mice, or malnourished (MN) mice treated IP with DMSO (vehicle control) or PGE2 synthase inhibitor (PGESi; CAY10526, 5mg/kg/day) starting 48h before the infection and evaluated at 48h p.i. *p = 0.04 (Kruskal-Wallis test). **B.** Percent of infected skin-derived neutrophils relative to total neutrophils in spleen of WN mice and MN mice treated IP with DMSO or MN mice treated with prostaglandin E2 synthase inhibitor (PGESi) (CAY10526, 5mg/kg/day). N = 5 mice per group.
(TIF)

S9 Fig Role of CCR7 in skin infection. **A**. CCR7 in BM monocytes transfected with control (scrambled) siRNA or siRNA targeting CCR7. Determined by qRT-PCR and expressed as fold-change over control(Left) or percentage of CCR7+ monocytes by flow cytometry (Right). ***p<0.0001 (Unpaired t-test). 4 replicates per group. **B.** BM derived monocytes transfected with CCR7-targeting siRNA or control siRNA, labeled and transferred ID to malnourished mice at the same time as infection ID with cell trace-labeled *L*. *donovani*. Percent of transferred monocytes in the skin (Left panel) and intensity of infection of transferred monocytes in the skin (right panel) determined by flow cytometry at 48h p.i. N = 10 mice per group. **C.** Proportion of Transferred monocytes (left panel) and intensity of the infection (right panel) in lymph node (LN) of malnourished mice recipients of monocytes transfected with CCR7-targeting siRNA or control siRNA. N = 9–10 per group. **D.** Percent of infected monocytes found in the lymph node (LN) of malnourished mice recipients of WT or CCR7KO donor monocytes 48 hr after of intradermal transfer and ID infection with mcherry-*L*. *donovani*. Donor cells identified with a Far-red cell trace and flow cytometry. Infected monocytes discriminated by the expression of Ly6C (Ly6C^int/hi^, inflammatory monocytes; Ly6C^low/-^, resident non-inflammatory monocytes). *p = 0.057 (Mann-Whitney test). N = 4 mice per group.
(TIF)
