# Supplementary material for: Malnutrition-related parasite dissemination from the skin in visceral leishmaniasis is driven by PGE2-mediated amplification of CCR7-related trafficking of infected inflammatory monocytes
Source: PLoS Negl Trop Dis. 2023 Jan 11;17(1):e0011040. doi: 10.1371/journal.pntd.0011040 (PMC9873180; doi:10.1371/journal.pntd.0011040)
Supplement: S1 Table — (DOCX) [file pntd.0011040.s001.docx]

**S1 Table. Antibodies and primers used in flow cytometry and qPCR assays**

| ***Target*** | ***Antibody*** | ***Clone*** |
| --- | --- | --- |
| *T cells* | CD3 | 17A2 |
| *B cells* | CD45R (B220) | Ra3-6B2 |
|  | CD19 | eBio 1D3 |
| *NK cells* | CD49b | DX5 |
|  | CD335 | 29A1.4 |
|  | NK-1.1 | PK136 |
| *Neutrophils* | Ly6G | 1A8 |
|  | Gr-1 (Ly6G/Ly6C) | RB6-8C5 |
| *DCs* | CD11c | N418 |
| *Monocytes* | CD11b | M1/70 |
|  | Ly6C | HK1.4 |
|  | CCR2 | 475301 |
| *CCR7* | CD197 | 4B12 |
| *CSF-1R* | CD115 | AFS98 |
| *CD45* | CD45 | 30-F11 |
|  |  |  |
| ***Target*** | **Primer Forward** | **Primer Reverse** |
| *Leishmania kDNA* | GGG TAG GGG CGT TCT GCG AA | GGC CCA CTA TAT TAC ACC ACC CCC |

Page Break
