# Supplementary material for: Malnutrition-related parasite dissemination from the skin in visceral leishmaniasis is driven by PGE2-mediated amplification of CCR7-related trafficking of infected inflammatory monocytes
Source: PLoS Negl Trop Dis. 2023 Jan 11;17(1):e0011040. doi: 10.1371/journal.pntd.0011040 (PMC9873180; doi:10.1371/journal.pntd.0011040)
Supplement: S2 Table — (DOCX) [file pntd.0011040.s002.docx]

**S2 Table. Percentage of monocytes in spleen of CSFR1-DTR mice treated with diphtheria toxin (DT) or PBS.** CSFR1-DTR mice were treated with DT to deplete CD115+ cells or were treated with PBS. Mice were left uninfected or were infected with *L.donovani* (72h).

| SPLEEN | Uninfected-DTR mice | | | | | | | |
| --- | --- | --- | --- | --- | --- | --- | --- | --- |
|  | **PBS** | | | | **DT** | | | |
|  | CD115- | | CD115+ | | CD115- | | CD115+ | |
|  | Mean | SEM | Mean | SEM | Mean | SEM | Mean | SEM |
| IMo (Ly6C^hi^) | 0.42 | 0.11 | 0.07 | 0.01 | 0.13 | 0.03 | 0.01 | 0.00 |
| IMo (Ly6C^int^) | 1.11 | 0.11 | 0.01 | 0.00 | 1.92 | 0.83 | 0.01 | 0.00 |
| R. Mo (Ly6C^low/-^) | 0.27 | 0.02 | 0.00 | 0.00 | 0.29 | 0.07 | 0.00 | 0.00 |
|  | **Infected-DTR mice** | | | | | | | |
|  | **PBS** | | | | **DT** | | | |
|  | CD115- | | CD115+ | | CD115- | | CD115+ | |
|  | Mean | SEM | Mean | SEM | Mean | SEM | Mean | SEM |
| IMo (Ly6C^hi^) | 0.51 | 0.07 | 0.07 | 0.02 | 0.15 | 0.03 | 0.01 | 0.00 |
| IMo (Ly6C^int^) | 1.62 | 0.16 | 0.01 | 0.00 | 1.37 | 0.24 | 0.01 | 0.00 |
| R. Mo (Ly6C^low/-^) | 0.40 | 0.03 | 0.00 | 0.00 | 0.26 | 0.03 | 0.00 | 0.00 |

Imo: Inflammatory monocyte; R. Mo: resident monocyte.
