# Supplementary figures and images for: Malnutrition-related parasite dissemination from the skin in visceral leishmaniasis is driven by PGE2-mediated amplification of CCR7-related trafficking of infected inflammatory monocytes
Source: PLoS Negl Trop Dis. 2023 Jan 11;17(1):e0011040. doi: 10.1371/journal.pntd.0011040 (PMC9873180; doi:10.1371/journal.pntd.0011040)

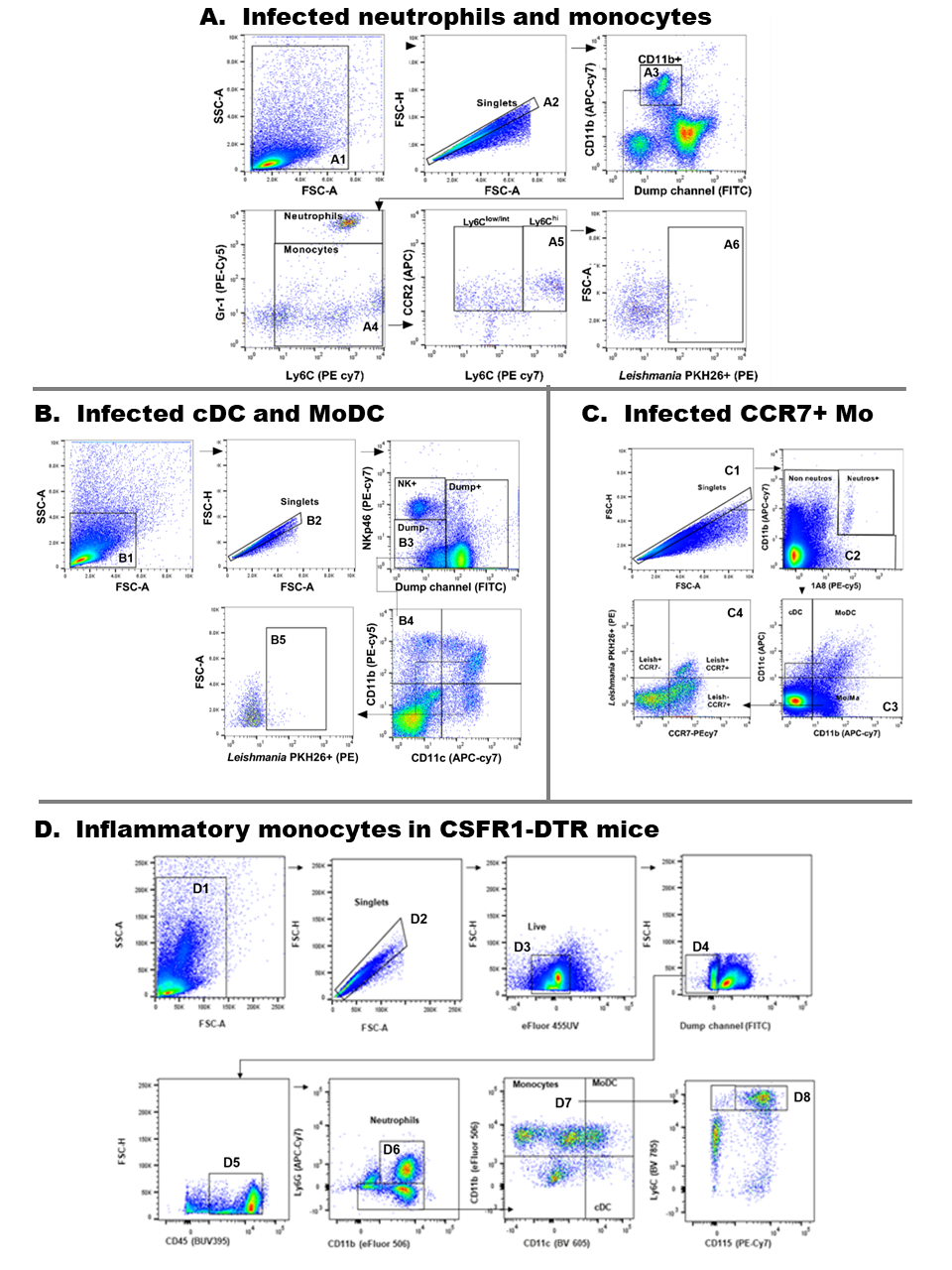

Supplement: S1 Fig — A. Gating strategy to identify parasite infected neutrophils and monocytes. Cells were identified by FSC and SSC properties (A1) and sub-gated in singlets by FSC-A vs. FSC-H (A2). Singlets were sub-gated to exclude dead cells, T cells (CD3+), and B cells (B220+), all in the FITC dump channel (A3). The CD11b+ (APC-cy7) population were sub-gated according to the expression of Ly6C+ (PE-Cy7) in monocytes (CD11b+Gr1low/-Ly6C+) or Gr-1 (PE-cy5) in neutrophils (Cd11b+Gr1hi Ly6C-/int) (A4). Monocytes were further classified according the expression of Ly6C as resident monocytes (Ly6Clow/-) or inflammatory monocytes (Ly6Chi+/int) (A5). All populations were sub-gated based the fluorescence of Leishmania labeled with PKH26 and FSC-A to identify infected cells (Leishmania PKH26+) (A6). B. Gating strategy to identify cDCs and MoDCs. Cells were identified by FSC and SSC properties (B1). Cells were sub-gated in singlets by FSC-A vs. FSC-H (B2). Singlets were sub-gated to exclude dead cells together with T cells (CD3+) and B cells (B220+) in the FITC dump channel (B3). After excluding NK cells (PE-cy7), cells were subgated to identify cDcs (CD11c APCcy7+ CD11b PEcy5-) and MoDcs (CD11c APC-cy7+ CD11b PE-cy5+) (B4). All populations were sub-gated based the fluorescence of the parasite labeled with PKH26 and FSC-A to identify infected cells (Leishmania PKH26+) (B5). C. Gating strategy to identify CCR7+ monocytes. After gating singlets in the cell population (C1) neutrophils were identified as Ly6G+CD11b+ cells (C2). Negative cells were sub-gated to identify dendritic cells (CD11c+CD11b-), monocyte-derived dendritic cells (CD11b+CD11c+) or monocytes (CD11b+CD11c-) (C3). Monocytes were sub-gated to identify CCR7+ Leishmania+ cells (C4). D. Gating strategy to identify inflammatory monocytes in CSFR1-DTR mice. Singlets were sub- gated as above (D1, D2). Dead cells (eF455 positive) were excluded (D3). T cells, NK cells were excluded in the dump channel (D4). Leukocytes (CD45+ positive) we [file pntd.0011040.s003.tif]

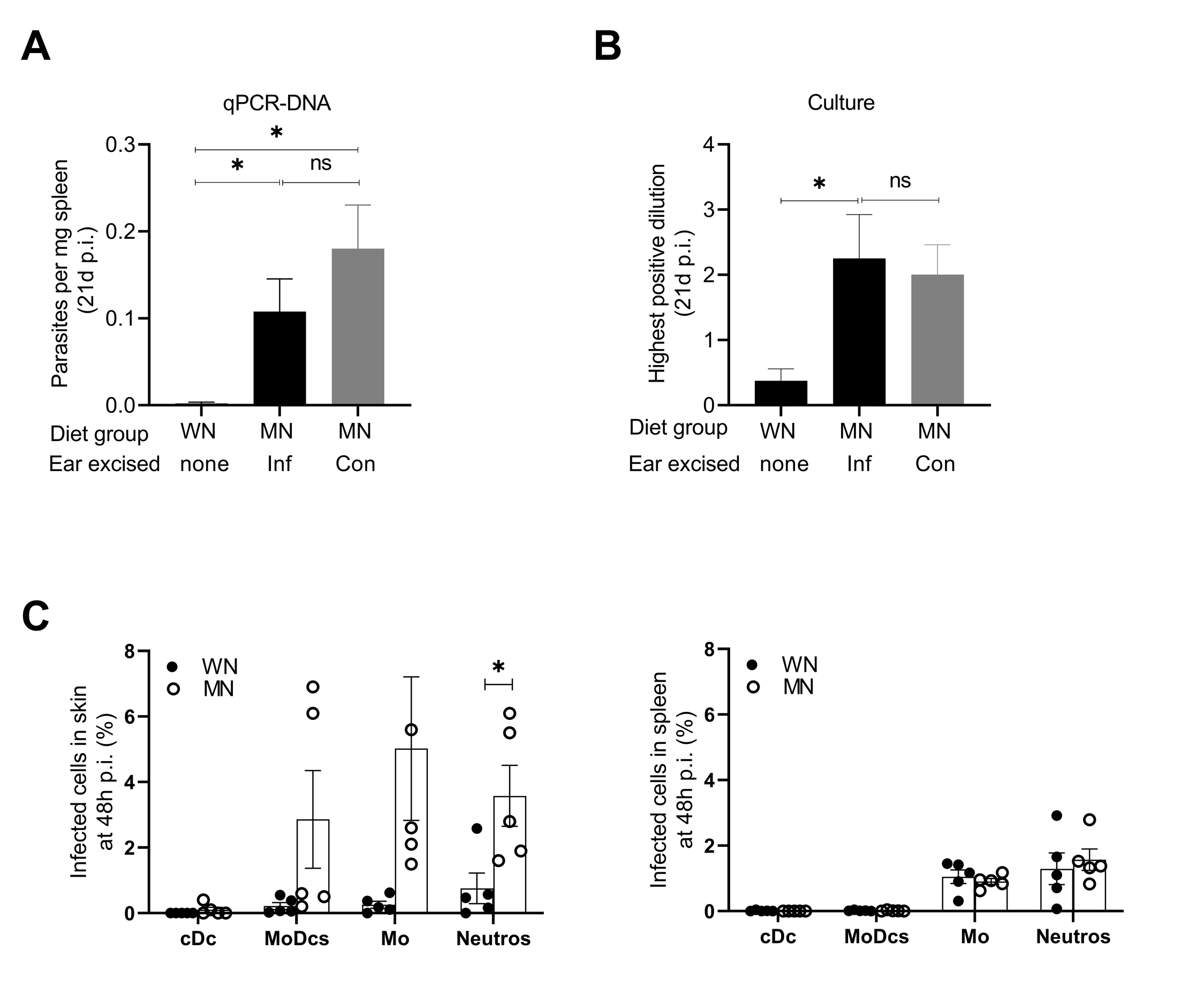

Supplement: S2 Fig — A,B. Visceralization of L. donovani in well-nourished (WN) mice or malnourished mice (MN) after early surgical excision of the infected ear pinna (Inf) or contralateral uninfected ear pinna (Con) at 72h p.i. Determined at 21 days after intradermal infection by expression of Leishmania kDNA in spleen tissue (qPCR) and Leishmania limiting-dilution culture. *p = 0.017; **p = 0.0021, (Kruskal-Wallis test). N = 8 mice per group. C. Percent of infected cells including conventional dendritic cells (cDC), monocyte derived dendritic cells (MoDCs), monocytes (Mo), and neutrophils (N) in skin and spleen at 48 hr post-infection relative to the total cells. Skin: *p = 0.03 (Mann-Whitney test, N = 5 mice per group). (TIF) [file pntd.0011040.s004.tif]

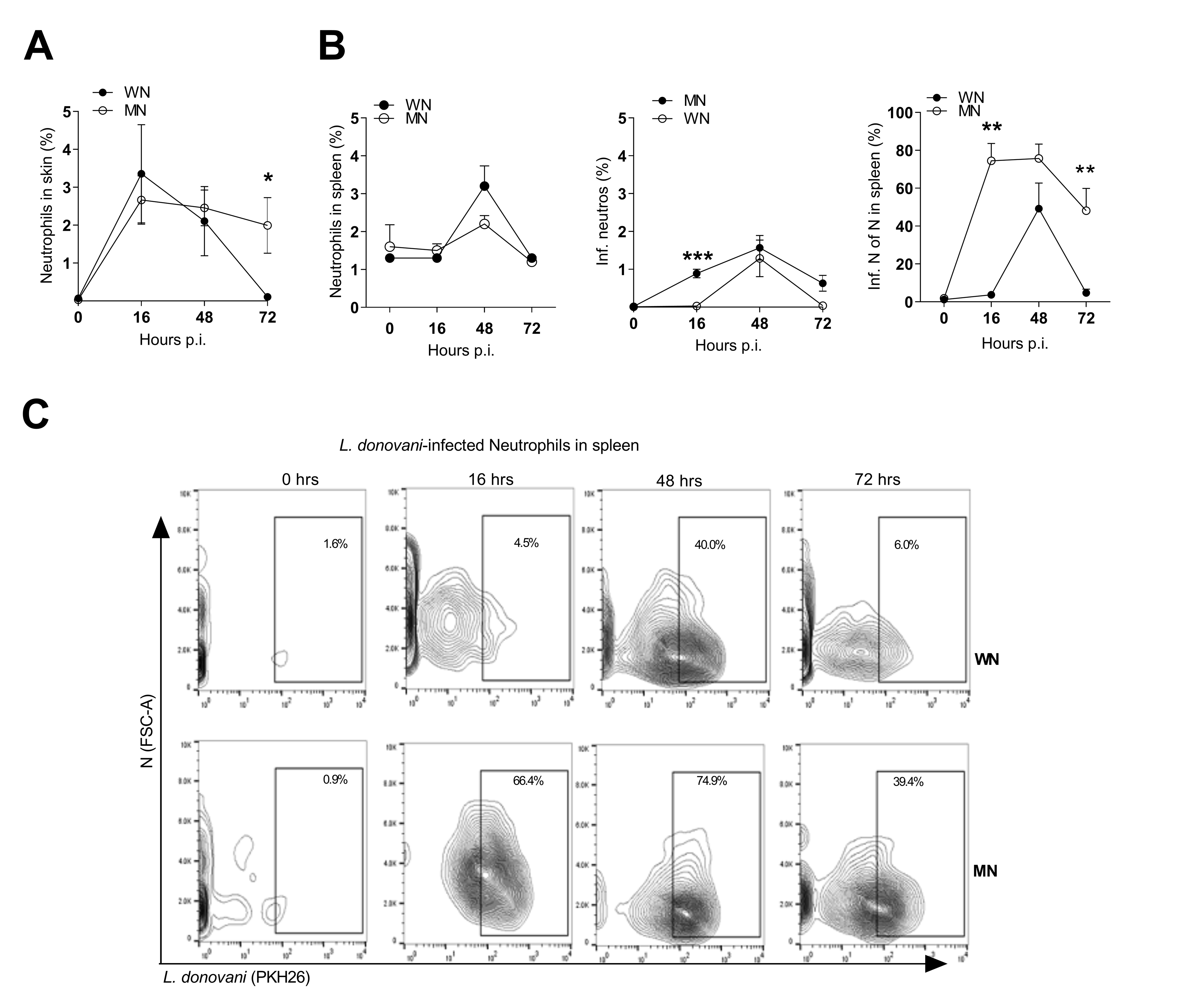

Supplement: S3 Fig — A. Kinetics of neutrophil accumulation in the skin of well-nourished (WN) or malnourished (MN) mice following intradermal L. donovani infection. *p = 0.033 (Unpaired t-test at 72h p.i.). N = 5 mice per group. B. Kinetics of neutrophil accumulation in the spleen of WN or MN mice presented as the percent of neutrophils relative to all spleen cells (Left panel), percent of infected neutrophils relative to all spleen cells (Middle panel), and percent of infected neutrophils relative to total splenic neutrophils (Right panel). *p = 0.040; **p = 0.014; ****p<0.0001 (2-way Anova). N = 5 mice per group. C. Representative contour plot gated in forward scatter (FSC) showing percent of Leishmania-infected neutrophils (CD11b+1A8+) relative to total splenic neutrophils. The full gating strategy is shown in S1A Fig. (TIF) [file pntd.0011040.s005.tif]

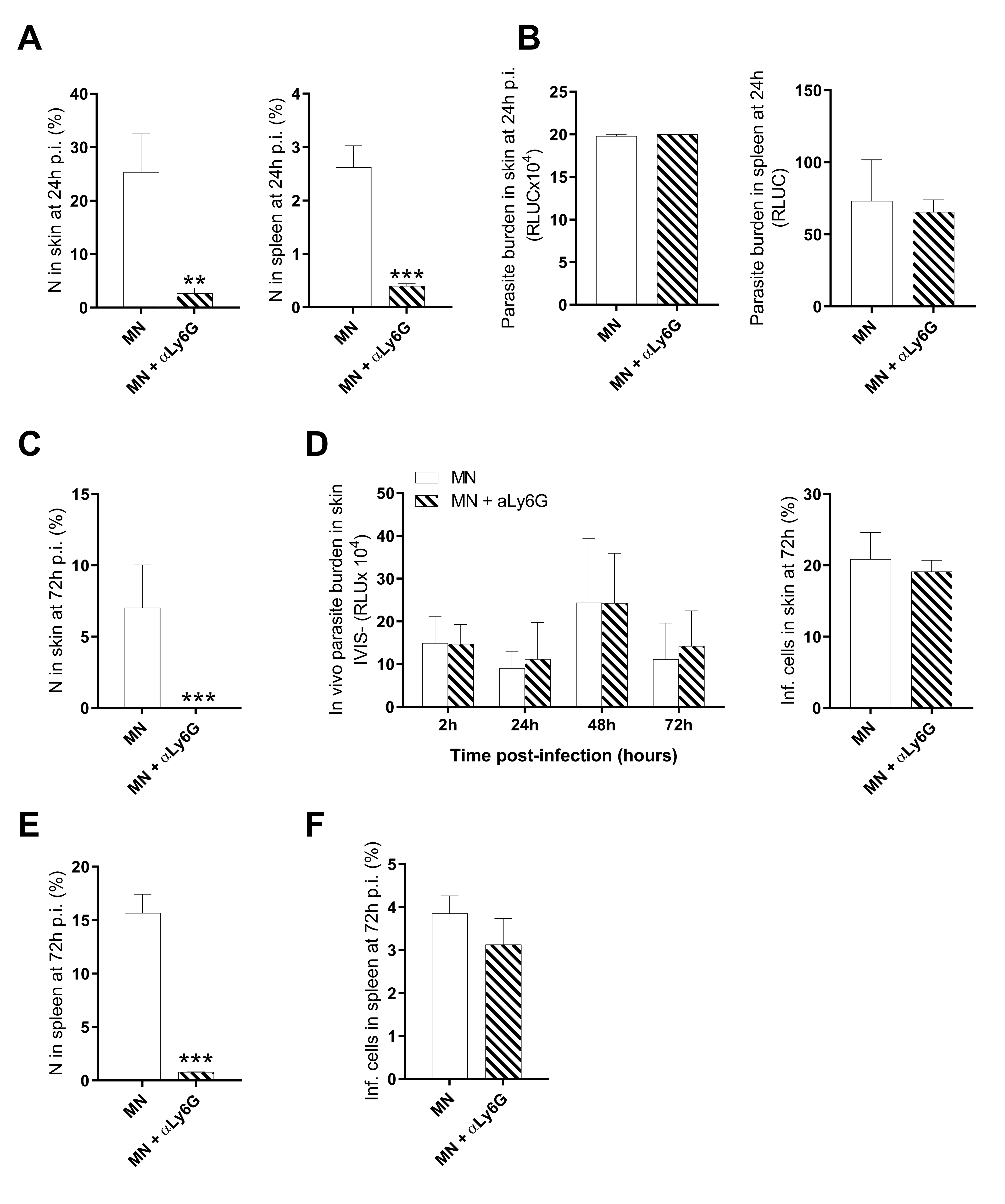

Supplement: S4 Fig — A. Reduced percent of neutrophils (N) in the skin and spleen (relative to total cells) of malnourished mice treated with the isotype control (MN) or with 200 μg of neutralizing anti-Ly6G antibody (MN+αLy6G) given by the intraperitoneal route the day before ID infection with L. donovani-LUC. Determined at 24 hrs p.i. **p = 0.014; ***p = 0.0006 (Unpaired t-test). B. Parasite burden in the skin infection site and spleen at 24h post-intection. Determined by luminometry. C. Reduced percent of neutrophils (N) in the skin (relative to total cells) of malnourished mice treated with the isotype control (MN) or with 200 μg of neutralizing anti-Ly6G antibody (MN+αLy6G) given by the intraperitoneal route the day before ID infection with L. donovani-LUC. Determined at 72 hrs p.i. ***p = 0.041 (Unpaired t-test). D. Left panel: Skin parasite burden in malnourished mice treated with the isotype control (MN) or with 200 μg of neutralizing anti-Ly6G antibody (MN+αLy6G) given by the intraperitoneal route the day before ID infection with L. donovani-LUC. Determined in vivo at 2–72 hrs p.i. Right panel: Percentage of infected cells found in skin after 72 hrs of infection and treatments as described above. E. Reduced percent of neutrophils in spleen (relative to toal cells) at 72h of ID infection with L. donovani. ***p = 0.0001 (Unpaired t-test). F. Percent of infected cells found in the spleen determined by flow cytometry. N = 5 malnourished mice per group. (TIF) [file pntd.0011040.s006.tif]

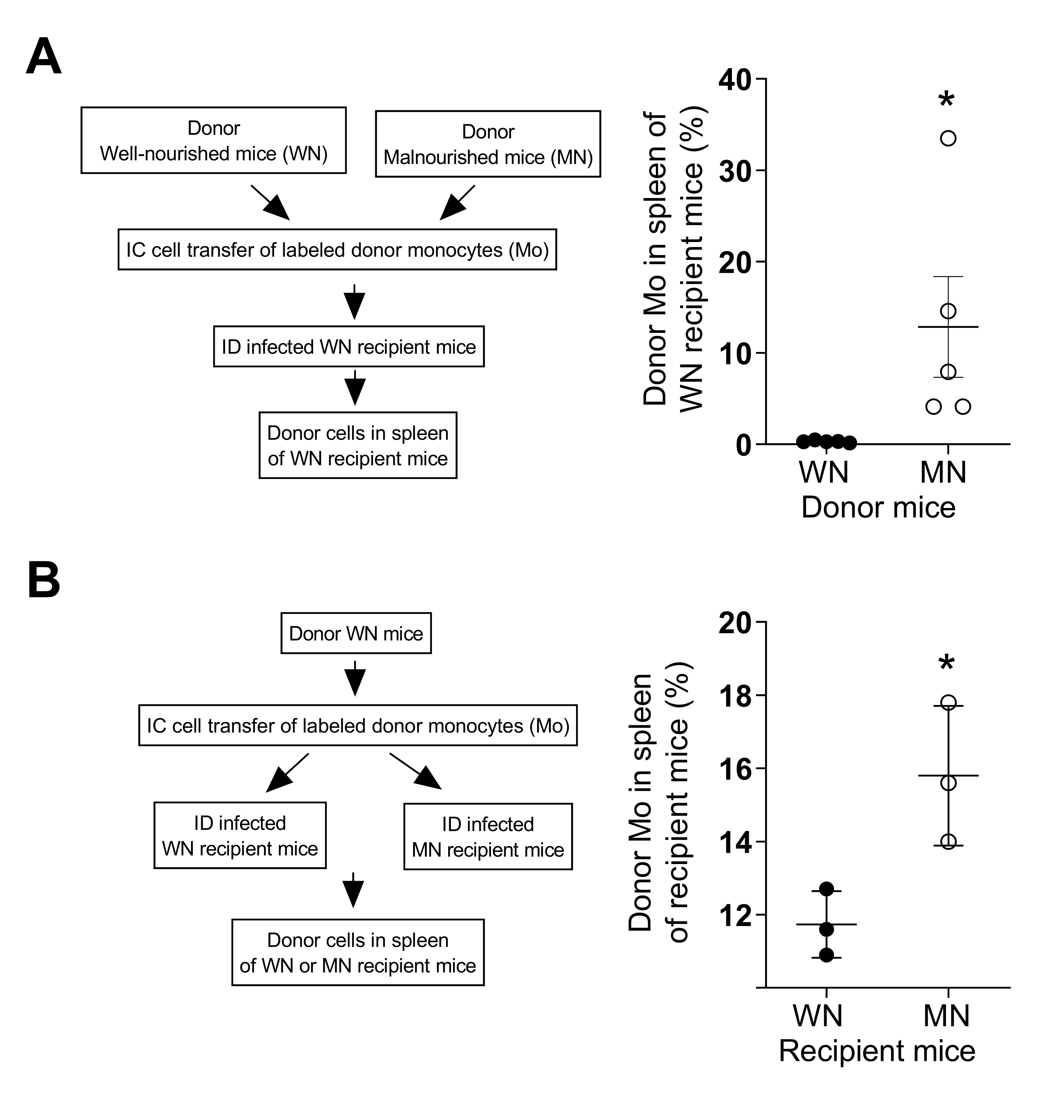

Supplement: S5 Fig — A. Schematic: bone marrow-derived monocytes from donor WN or MN mice were labeled with PKH-67 or PKH-26, combined in equal numbers, and transferred by intracardial (IC) injection to recipient control mice infected ID at the same time with L. donovani. Labeled monocytes that trafficked to spleen after IC delivery were enumerated by flow cytometry and data are expressed as percent of labeled monocytes relative to total splenic monocytes. *p = 0.052 (Unpaired t-test). N = 5 donor mice per group and 5 recipient mice. B. Schematic: bone marrow-derived monocytes from donor well-nourished control mice were labeled and transferred IC to recipient WN or MN mice infected ID at the same time with L. donovani. Labeled monocytes that trafficked to spleen after IC delivery were enumerated by flow cytometry and data are expressed as percent of labeled monocytes relative to total splenic monocytes. Data shown are from a single experiment that is representative of two independent experiments using 3–5 mice per group. *p = 0.029 (Unpaired t-test). N = 5 donor mice and 3 recipient mice per group. (TIF) [file pntd.0011040.s007.tif]

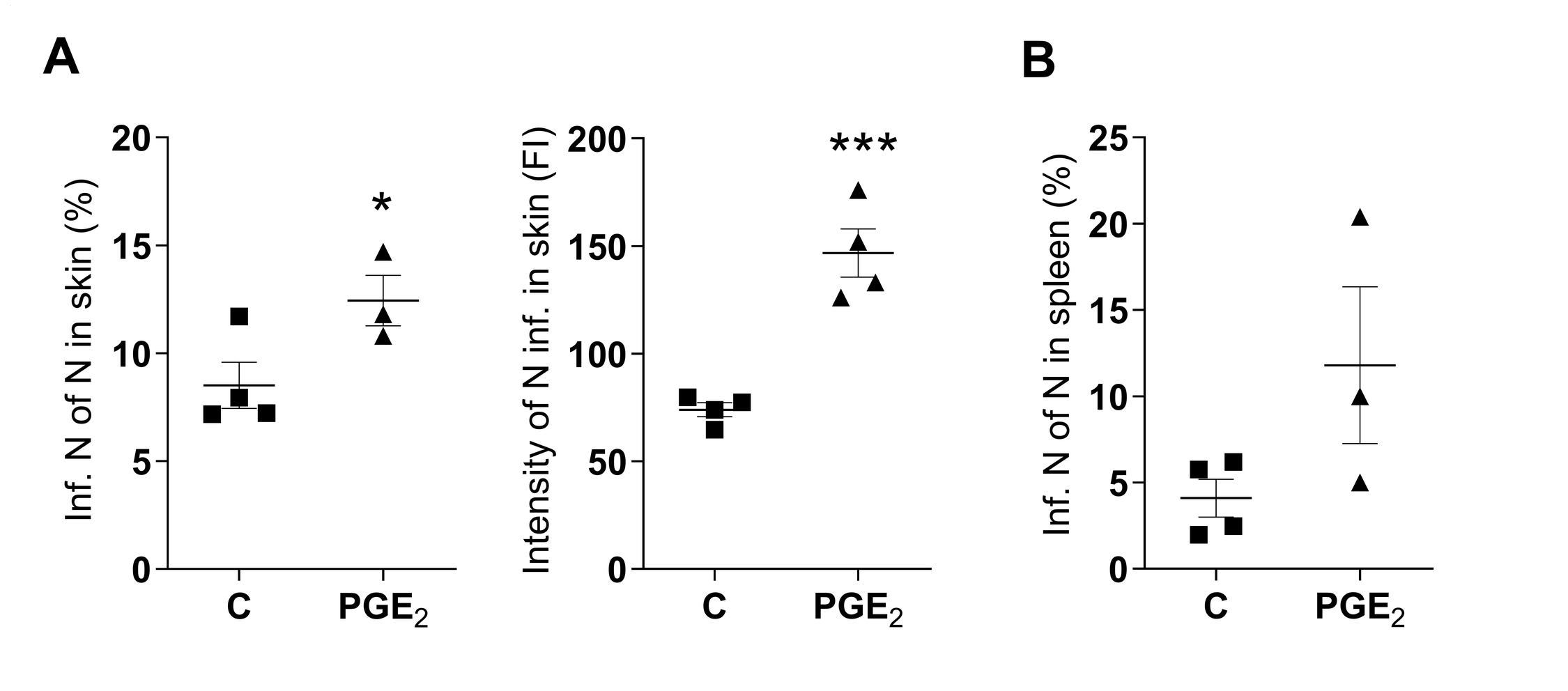

Supplement: S6 Fig — A. Percent of infected neutrophils (N) (Left panel), and intensity of infection (fluorescence intensity of parasites; right panel) of neutrophils in mice injected ID with DMSO (vehicle control; C) or PGE2 (10 ng in DMSO) followed immediately by ID infection with fluorescent-L. donovani. *p = 0.058; ***p = 0.0008 (Unpaired t-test). B. Percent of infected neutrophils in spleen of intradermally infected control mice (C) or mice treated ID with 10 ng PGE2 at the time of ID infection. Evaluated at 48h p.i. by flow cytometry. N = 4 mice per group. (TIF) [file pntd.0011040.s008.tif]

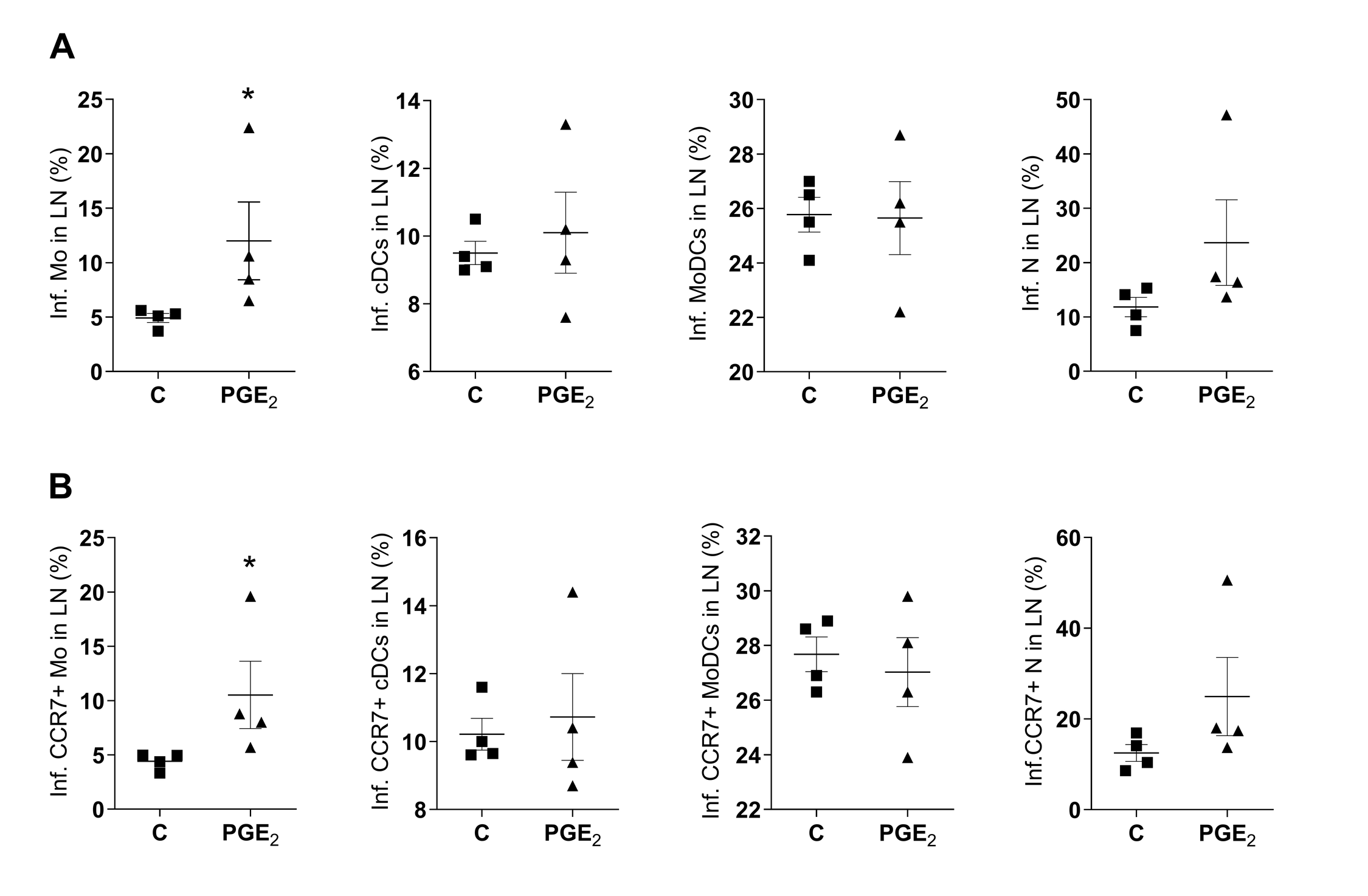

Supplement: S7 Fig — A. Proportion of infected monocytes (Mo), conventional dendritic cells (cDCs), monocyte derived dendritic cells (moDCs) and neutrophils in lymph node (LN) of DMSO (C; vehicle control) or PGE2 (10 ng in DMSO) in ID treated mice at the time of ID infection with fluorescent-L. donovani. *p = 0.028 (Mann-Whitney test). B. Proportion of infected CCR7-expressing monocytes, cDCs, MoDcs, and neutrophils in LN of DMSO (C; vehicle control) or PGE2 (10 ng in DMSO) in ID treated mice at the time of ID infection with fluorescent-L. donovani. Evaluated by flow cytometry at 48h post-infection. Representative of 2 independent experiments. *p = 0.028 (Mann-Whitney test). N = 4 mice per group. (TIF) [file pntd.0011040.s009.tif]

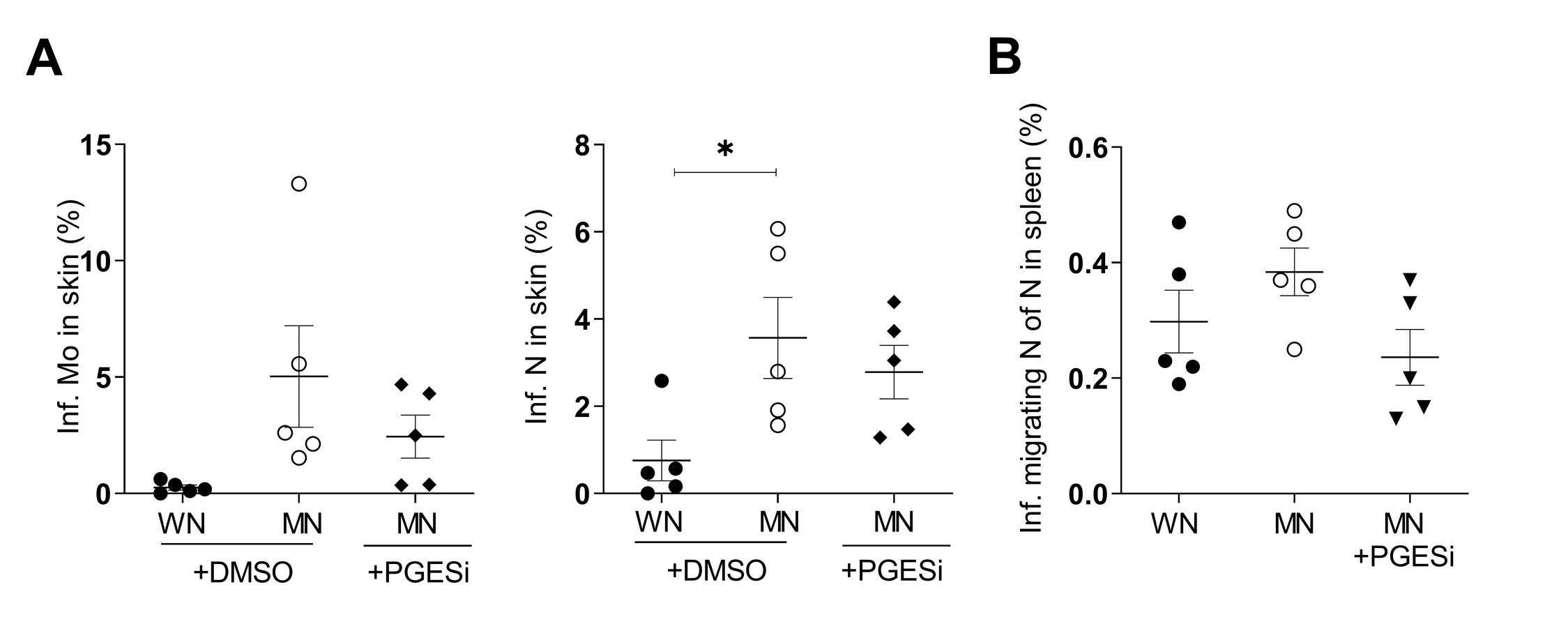

Supplement: S8 Fig — A. Proportion of infected monocytes and infected neutrophils in skin of well-nourished (WN) mice, or malnourished (MN) mice treated IP with DMSO (vehicle control) or PGE2 synthase inhibitor (PGESi; CAY10526, 5mg/kg/day) starting 48h before the infection and evaluated at 48h p.i. *p = 0.04 (Kruskal-Wallis test). B. Percent of infected skin-derived neutrophils relative to total neutrophils in spleen of WN mice and MN mice treated IP with DMSO or MN mice treated with prostaglandin E2 synthase inhibitor (PGESi) (CAY10526, 5mg/kg/day). N = 5 mice per group. (TIF) [file pntd.0011040.s010.tif]

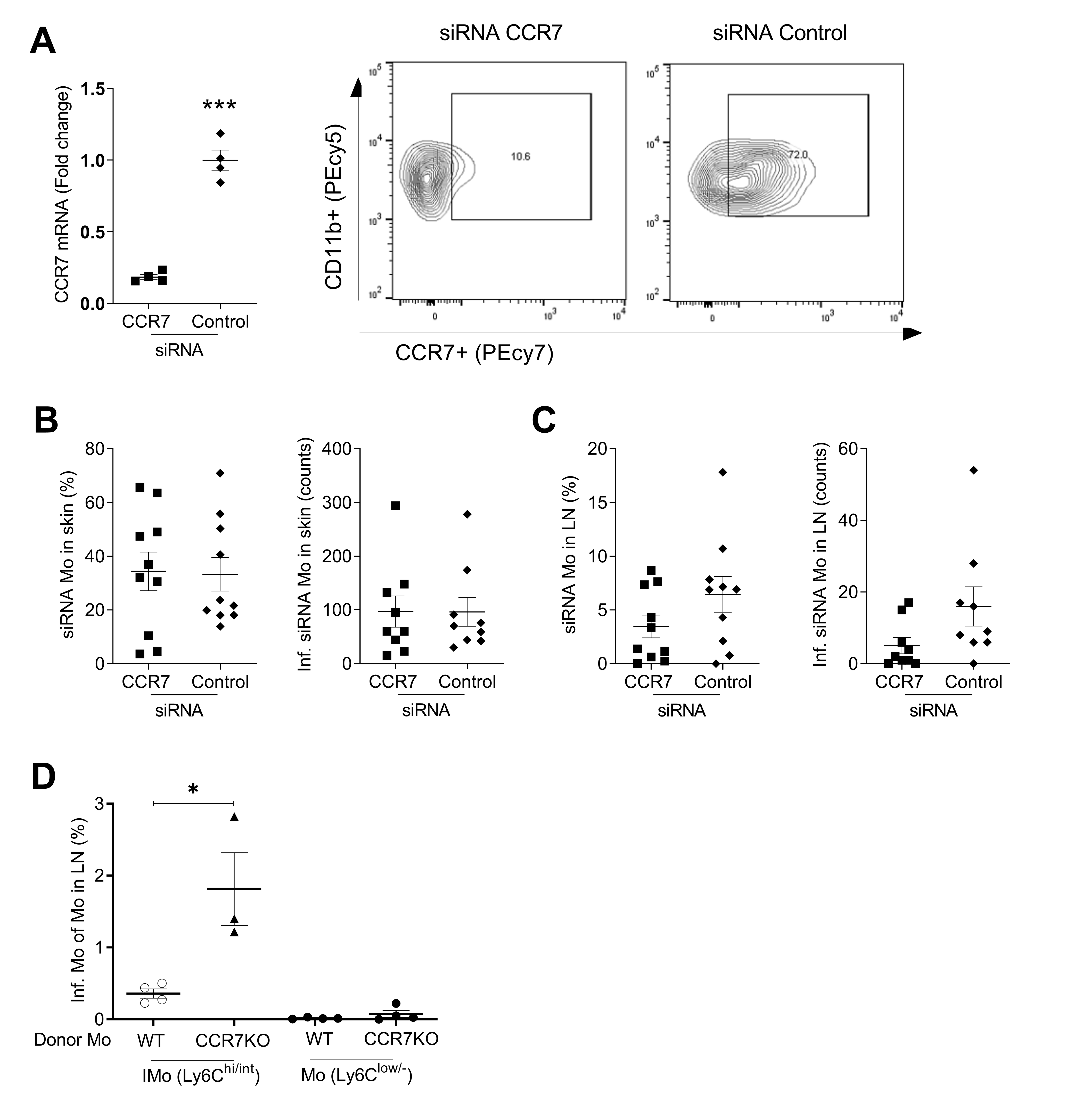

Supplement: S9 Fig — A. CCR7 in BM monocytes transfected with control (scrambled) siRNA or siRNA targeting CCR7. Determined by qRT-PCR and expressed as fold-change over control(Left) or percentage of CCR7+ monocytes by flow cytometry (Right). ***p<0.0001 (Unpaired t-test). 4 replicates per group. B. BM derived monocytes transfected with CCR7-targeting siRNA or control siRNA, labeled and transferred ID to malnourished mice at the same time as infection ID with cell trace-labeled L. donovani. Percent of transferred monocytes in the skin (Left panel) and intensity of infection of transferred monocytes in the skin (right panel) determined by flow cytometry at 48h p.i. N = 10 mice per group. C. Proportion of Transferred monocytes (left panel) and intensity of the infection (right panel) in lymph node (LN) of malnourished mice recipients of monocytes transfected with CCR7-targeting siRNA or control siRNA. N = 9–10 per group. D. Percent of infected monocytes found in the lymph node (LN) of malnourished mice recipients of WT or CCR7KO donor monocytes 48 hr after of intradermal transfer and ID infection with mcherry-L. donovani. Donor cells identified with a Far-red cell trace and flow cytometry. Infected monocytes discriminated by the expression of Ly6C (Ly6Cint/hi, inflammatory monocytes; Ly6Clow/-, resident non-inflammatory monocytes). *p = 0.057 (Mann-Whitney test). N = 4 mice per group. (TIF) [file pntd.0011040.s011.tif]
